# Supplementary material for: Development of a human iPSC-derived corticospinal tract-on-a-chip
Source: Cell Rep Methods. 2026 May 19;6(7):101457. doi: 10.1016/j.crmeth.2026.101457 (PMC13390070; doi:10.1016/j.crmeth.2026.101457)
Supplement: Document S1. Figures S1–S3 [file mmc1.pdf]

Cell Reports Methods, Volume *6*

Supplemental information

Development of a human iPSC-derived corticospinal tract-on-a-chip

Andriana Charalampopoulou, Arens Taga, Khalil Rust, Evelyn Luciani, Katherine Marshall, Elliot Montgomery, Anuradha Mansinghka, Richa Singh, Yang Zhao, Christine O'Keefe, Tza-Huei Wang, Arun Venkatesan, Christa Whelan Habela, and Nicholas John Maragakis

A *Addition of live cell dye at the cortical compartment only*

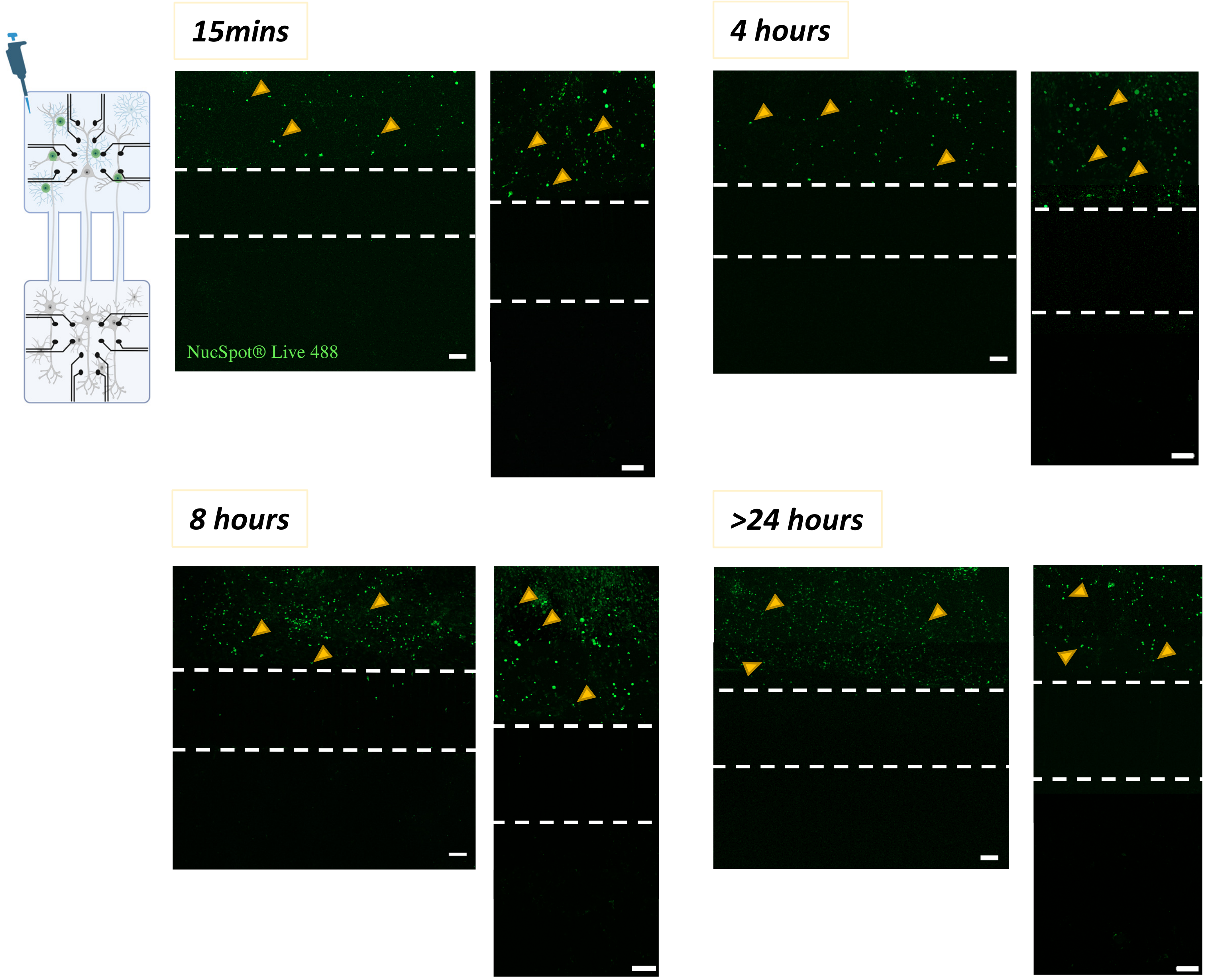

B *Subsequent addition at the spinal cord compartment*

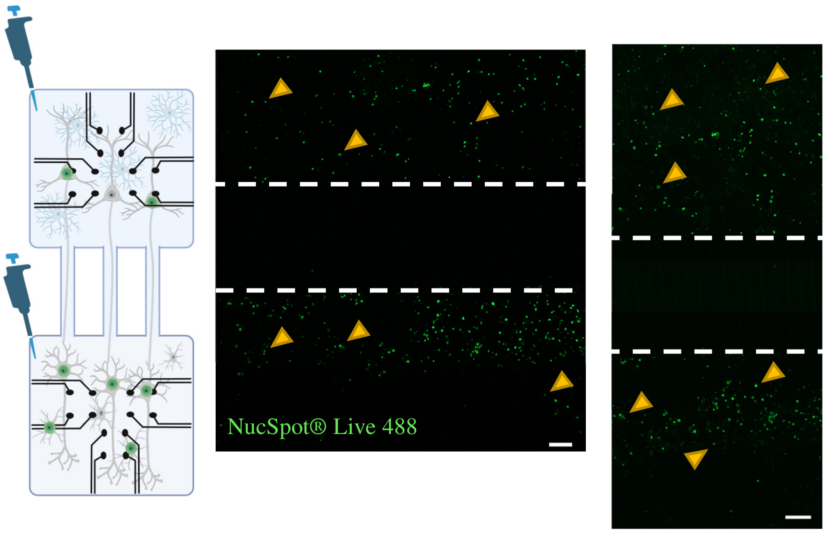

Supplemental Figure 1: Live cell diffusion demonstrates fluidic isolation between compartments related to STAR Methods.

A. Time-lapse imaging of dye diffusion in the microfluidic device following the addition of a live-cell dye to the cortical compartment. Mosaic assembled manually from overlapping confocal fields to represent the broader field of view. Images were captured at 15 minutes, 4 hours, 8 hours, and >24 hours post-addition to monitor diffusion dynamics across the microchannels (n=3 per condition). **B.** Representative mosaic live-cell images of microfluidic devices after dye addition to both compartments, demonstrating expected diffusion patterns (n=3). Scale bars=100μm.

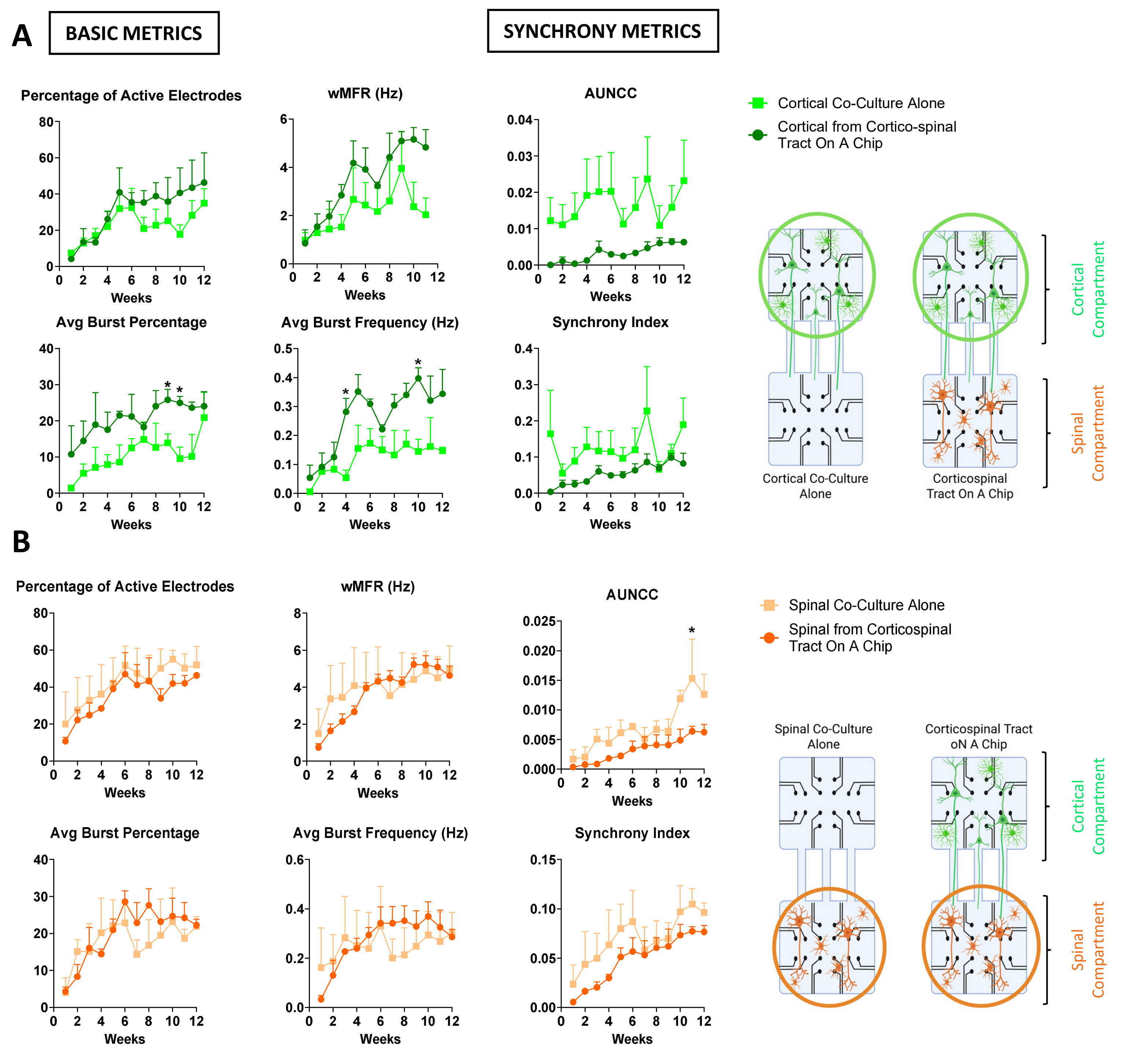

Supplemental Figure 2: Comparison of electrophysiological activity and maturation of single population chips (one neuronal population) with CST-on-a Chip (two neuronal populations), related to Figure 4.

A. CNs and CA single population cultures compared to CST-On-A-Chip setup with both cortical and spinal co-cultures **B.** SpMN and SpA compared to CST-On-A-Chip setup, and illustration of both plating setups (n=8-9 devices on MEA per condition). One-way ANOVA, * $p<0.05$, wMFR: weighted mean firing rate, AUNCC: area under normalized cross correlation.

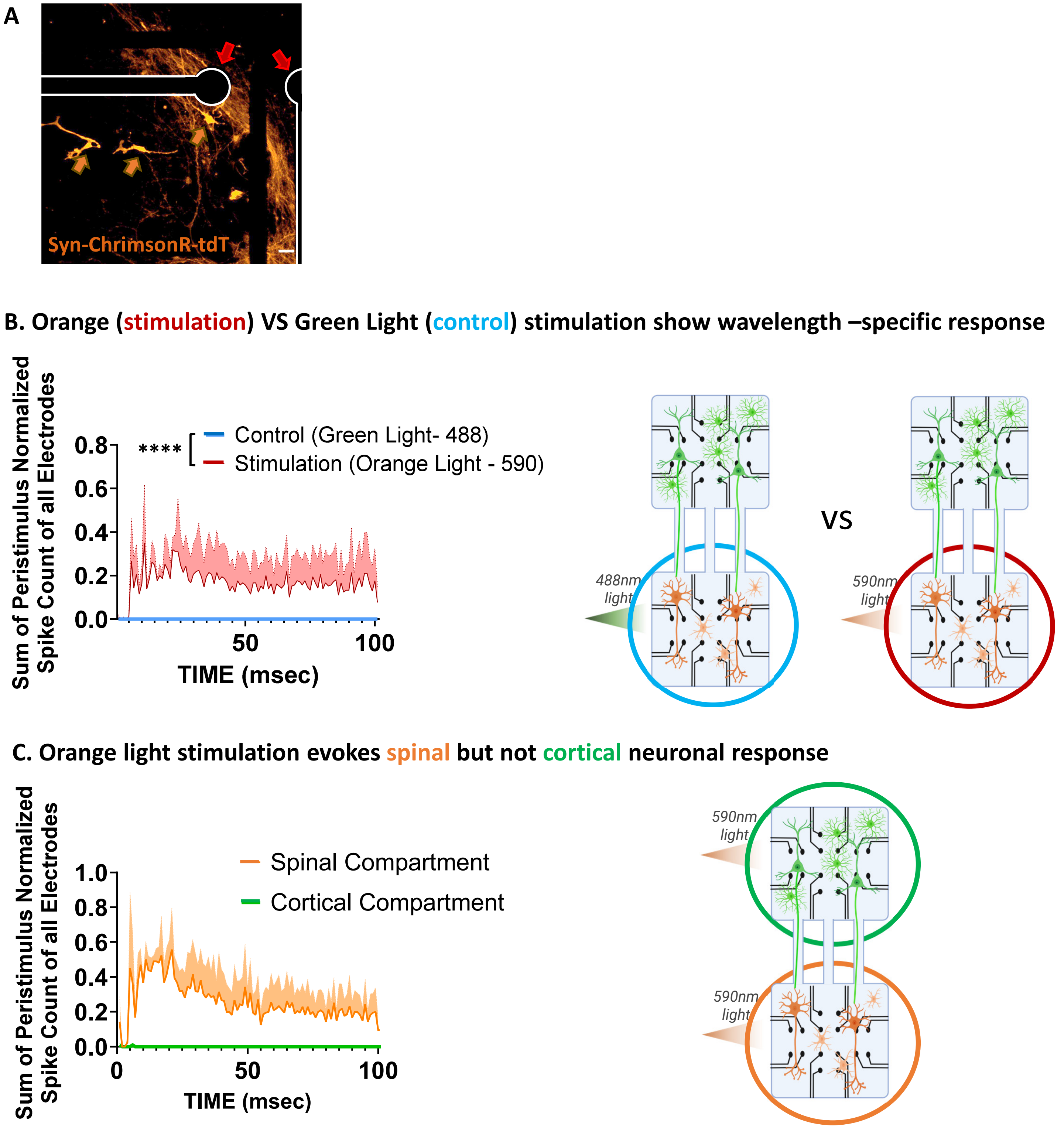

Supplemental Figure 3: Optogenetic activation of SpMN confirms absence of retrograde cortical electrophysiological responses, related to Figure 6.

A. Live Fluorescence image of td Tomato expressed under the synapsin promoter in the spinal compartment (red arrows show electrodes, orange arrows show transduced spinal neurons). **B.** The response recorded from the spinal neurons is specific to orange light stimulation (ChrimsonR-related response), and is significantly higher compared to the control stimulation (green light). **C.** Orange light stimulation activates spinal cord neurons transduced with AAV9-Syn-ChrimsonR-tdT whereas there is no corresponding response from the cortical neurons. Colored circles represent chambers from which data were recorded. Wilcoxon test, **** P<0.0001. Scale bar 50μm.
