# Supplementary material for: Development of a human iPSC-derived corticospinal tract-on-a-chip
Source: Cell Rep Methods. 2026 May 19;6(7):101457. doi: 10.1016/j.crmeth.2026.101457 (PMC13390070; doi:10.1016/j.crmeth.2026.101457)
Supplement: Document S3. Article plus supplemental information [file mmc3.pdf]

Cell Reports Methods

Article

Development of a human iPSC-derived corticospinal tract-on-a-chip

Graphical abstract

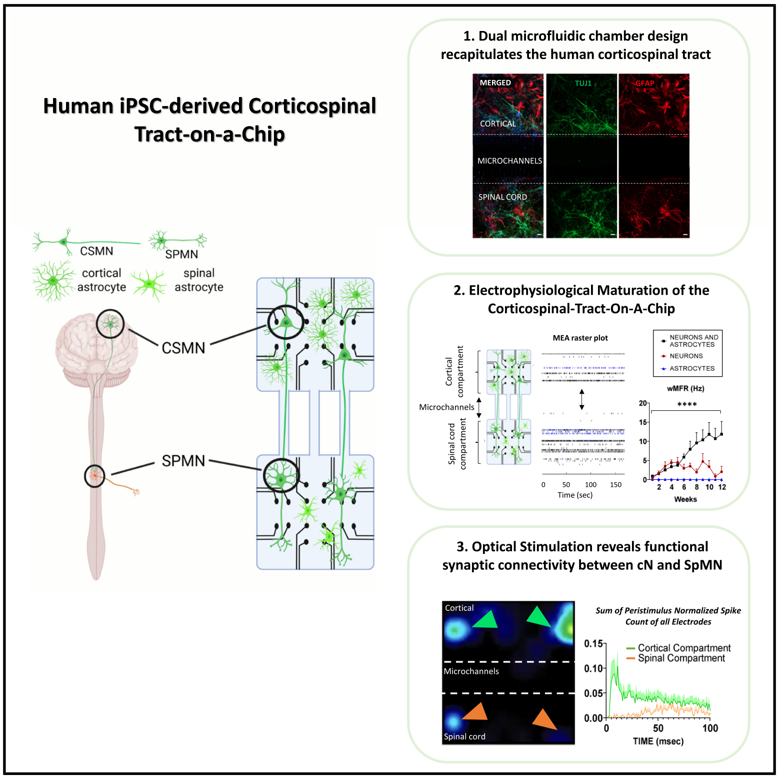

Highlights

- hiPSC microfluidic platform models human corticospinal circuits for disease research
- The platform includes cortical and spinal astrocytes with CNs and SpMNs
- MEA shows network maturation and retrograde tracing confirm CN-SpMN synapses
- Optogenetic CN activation drives spinal activity, validating function

Authors

Andriana Charalampopoulou, Arens Taga, Khalil Rust, ..., Arun Venkatesan, Christa Whelan Habela, Nicholas John Maragakis

Correspondence

nmaragak@jhmi.edu

In brief

Charalampopoulou et al. establish a human corticospinal tract-on-a-chip by integrating hiPSC-derived cortical and spinal motor neurons with region-matched astrocytes in a compartmentalized microfluidic MEA platform. The model supports long-term maturation, retrograde verification of corticospinal connectivity, and optogenetic activation of excitatory pathways that are sensitive to glutamatergic antagonists. This system enables longitudinal, compartment-specific study of corticospinal circuits and translational screening.

Charalampopoulou et al., 2026, Cell Reports Methods *6*, 101457
July 20, 2026 © 2026 The Authors. Published by Elsevier Inc.
https://doi.org/10.1016/j.crmeth.2026.101457

CellPress

Cell Reports Methods

CellPress
OPEN ACCESS

Article

Development of a human iPSC-derived corticospinal tract-on-a-chip

Andriana Charalampopoulou,[1,3] Arens Taga,[1] Khalil Rust,[1] Evelyn Luciani,[1] Katherine Marshall,[1] Elliot Montgomery,[1] Anuradha Mansinghka,[1] Richa Singh,[1] Yang Zhao,[2] Christine O'Keefe,[2] Tza-Huei Wang,[2] Arun Venkatesan,[1] Christa Whelan Habela,[1] and Nicholas John Maragakis[1,3,*]

[1]Department of Neurology, Johns Hopkins University, Baltimore, MD 21205, USA

[2]Department of Biomedical Engineering, Johns Hopkins University, Baltimore, MD 21205, USA

[3]Lead contact

*Correspondence: nmaragak@jhmi.edu

https://doi.org/10.1016/j.crmeth.2026.101457

MOTIVATION Degeneration of the corticospinal tract is a key feature of numerous neurodegenerative diseases, yet current *in vitro* models lack the anatomical and functional fidelity to study this system. We developed a human induced pluripotent stem cell-derived corticospinal tract-on-a-chip using a multielectrode array platform that incorporates regionally patterned cortical and spinal neurons and astrocytes. This model demonstrates structural and functional synaptic connectivity and enables longitudinal electrophysiological recordings. Critically, it supports compartment-specific manipulation and real-time analysis of corticospinal tract network dynamics, capabilities lacking in existing systems.

SUMMARY

Degeneration of the corticospinal tract is a feature in several neurodegenerative disorders and leads to disability. However, modeling corticospinal neuron (CSN) pathology and corticospinal connectivity is challenging, as there are interspecies differences in these networks. We developed a human induced pluripotent stem cell (hiPSC)-based microfluidic platform for modeling human CSN and spinal motor neuron (SpMN) connectivity. The incorporation of regionally specific astrocyte subtypes (cortical and spinal) in addition to CSNs and SpMNs allows for the modeling of neural cell interactions. Multielectrode array electrophysiology reveals the temporal maturation of the network. Retrograde labeling demonstrates synaptic connectivity between CSNs and SpMN. Optogenetic strategies to selectively activate excitatory cortical neurons (CNs) attenuated by glutamate receptor antagonism confirm the functional relevance of the model. Incorporating morphological, electrophysiological, and physiological measures of corticospinal connectivity, this platform is a versatile model for neurodegenerative disease research and the future development of targeted CSN therapies.

INTRODUCTION

Corticospinal tract (CST) dysfunction is a hallmark of several neurodegenerative disorders, including amyotrophic lateral sclerosis (ALS), primary lateral sclerosis, primary progressive multiple sclerosis (PPMS), and hereditary spastic paraplegia (HSP).[1] The pathophysiology of these conditions is marked by the degeneration of long-projecting cortical neurons (CNs), or "dying-back" axonal degeneration, ultimately leading to secondary neuronal cell death. Additionally, the corticospinal tract has been hypothesized to serve as a conduit for disease propagation in neurodegenerative disorders, driven by prion-like protein aggregation and functional disruptions through neuronal hyper- or hypo-excitability.[2,3] This is prototypically exemplified in ALS, where motor neuron degeneration is hypothesized to begin focally in either the cortex or spinal cord and spreads both locally, affecting adjacent motor neurons, and through the corticospinal tract, affecting distant cortical and spinal motor neuron (SpMN) populations.[3] One proposed mechanism underlying this spread is the "corticofugal hypothesis," which posits a directional propagation of pathology from cortical upper motor neurons to their downstream targets in the brainstem and spinal cord via the corticospinal tract. However, evidence also suggests the potential for bidirectional and multisite onset, indicating a more complex pattern of disease dissemination.

Modeling the corticospinal tract in normal conditions in animal models presents significant challenges due to species-specific differences in the organization and connectivity of descending corticospinal axons.[4] For example, in rodents, the majority of corticospinal projections terminate on spinal interneurons, with minimal direct input to alpha motor neurons. In contrast, primates, and especially humans, exhibit a greater proportion of

© 2026 The Authors. Published by Elsevier Inc.
This is an open access article under the CC BY license (http://creativecommons.org/licenses/by/4.0/).

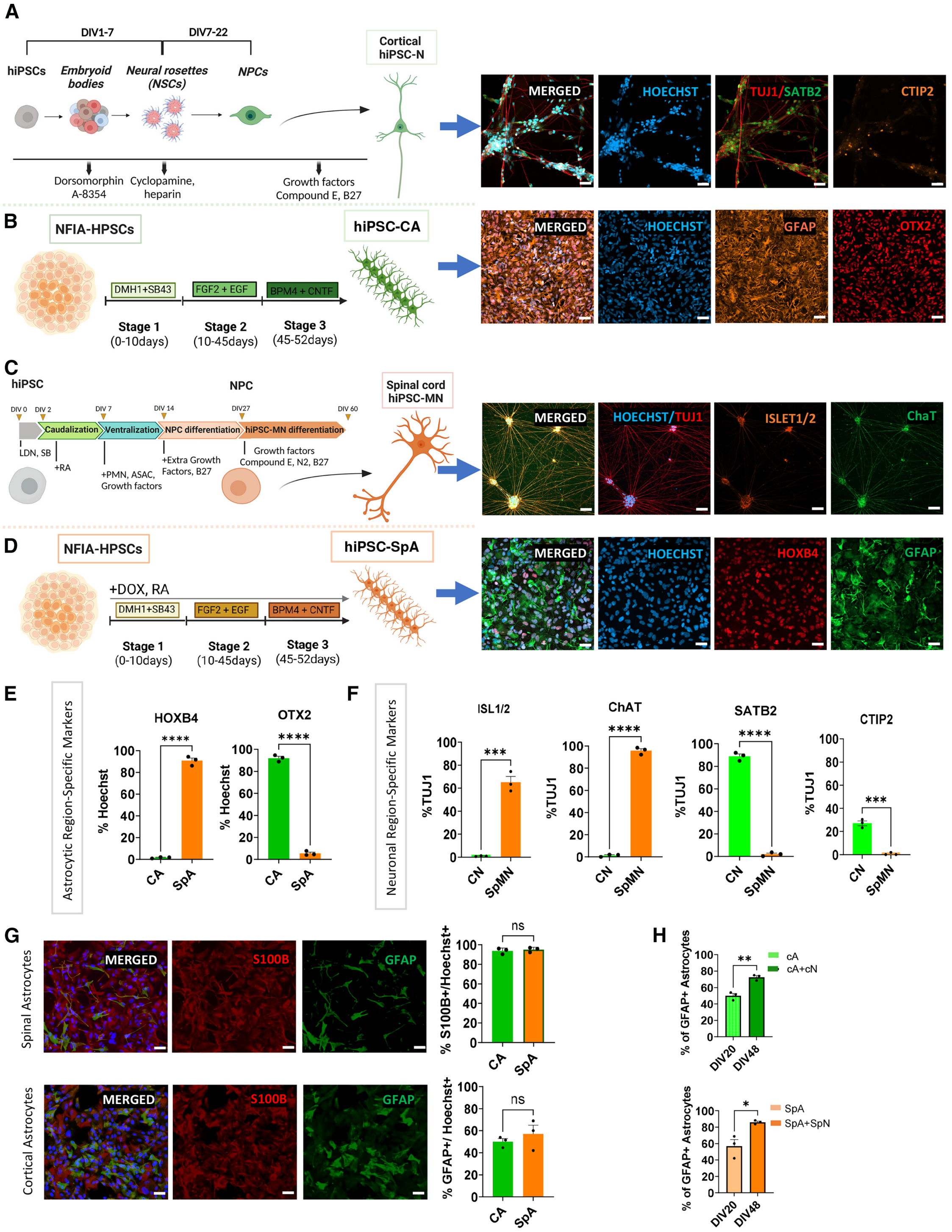

Figure 1. Differentiation protocols yield regionally specific cortical and spinal cord neurons and astrocytes

(A and B) (A) Schematic diagram for generation of cortical neurons from hiPSC and (B) diagram of hiPSC-derived cortical astrocyte differentiation of BrainXell astrocytes (modified BrainXell illustration) with representative images of immunostaining for region-specific markers.

(C) Schematic diagram for generation of SpMNs from hiPSCs and (D) diagram of hiPSC-derived spinal astrocyte differentiation of BrainXell astrocytes (modified BrainXell illustration) with representative immunostaining images showing the region-specific markers.

(E) Quantification of region-specific astrocytic markers performing immunohistochemistry showing strong regional identity as indicated by high OTX2 expression in the cortical astrocytes and high HOXB4 expression in the spinal astrocytes.

(legend continued on next page)

corticospinal axons that form direct monosynaptic connections with SpMNs, most notably those innervating distal limb muscles responsible for fine motor function.[5] This includes projections from large layer V pyramidal neurons (or Betz cells) in the motor cortex, which are uniquely prominent in humans. Under disease conditions, traditional animal models of disorders involving corticospinal dysfunction have historically been utilized to provide insights into potential pathogenic mechanisms.[6] However, these models also introduce significant interspecies differences that may limit their translational relevance, particularly for therapeutic applications.[7] In ALS, for example, mouse models have been instrumental in demonstrating early degeneration of corticospinal neurons (CSNs), supporting the hypothesis of corticofugal disease propagation[8,9] and exploring neuroprotective strategies to promote CSN survival.[10–13] However, species-specific differences in the connectivity between CSNs and SpMNs may affect the extent of CSN-related pathology in these models.[4,14–16] In diseases that affect both ends of the corticospinal pathway—as in ALS, where dysfunction of both CSNs and SpMNs is evident—the relative clinical and electrophysiological accessibility of assessing SpMN dysfunction, combined with its prominent involvement, has historically biased animal models toward a focus on SpMNs.[17] Consequently, interventions that appeared effective or promising in preclinical animal models have systematically failed to demonstrate efficacy in human clinical trials, highlighting a persistent and frustrating barrier to successful therapeutic translation.[18]

The use of human induced pluripotent stem cells (hiPSCs) has emerged as a promising approach to overcome the limitations of animal models, including the ability to study diseases that lack specific gene mutations as causative events. Furthermore, to approximate central nervous system modeling, the incorporation of other non-neuronal cell types into culture systems, whether in 3D organoids or 2D co-cultures,[19] has demonstrated significant enhancements in neuronal maturation, complexity, and network formation,[20] increasing the physiological relevance of these models. In translational studies, co-culturing neurons with glial cells has enabled the modeling of key pathological features, such as TDP-43 mislocalization and axonal degeneration observed in ALS, HSP, and MS.[21–24] Two-dimensional co-cultures, in particular, offer advantages in terms of controlled culture conditions and cellular composition, enabling the integration of patient-derived cells and the evaluation of cell-autonomous or non-cell-autonomous roles of specific cell types.[25,26] These systems have been effectively utilized to model local cortical and spinal cord networks, as well as motor neuron-muscle interactions.[27–29]

Despite these advancements, the development of *in vitro* models to study the corticospinal tract remains largely underexplored. Previously reported systems have lacked anatomical relevance and the versatility needed for reliable translational outcomes.[27,30,31] To address these limitations, we have developed a corticospinal tract-on-a-chip model that offers a unique set of advantages. The platform uses microfluidic chambers to spatially isolate hiPSC-derived cortical and spinal neurons and astrocytes to generate region-specific co-cultures. This platform mimics the anatomical organization of the human corticospinal tract, enabling the study of region-specific neuronal and astrocyte interactions as well as synaptic transmission. The microfluidic separation of individual cultures and corticospinal axons enables selective manipulation of each compartment. By incorporating optogenetic strategies for activating specific neuronal subpopulations and electrophysiological analyses with multielectrode array (MEA), this approach allows for the assessment of the dynamic properties of synaptic connectivity and network activity. This platform establishes a distinct experimental framework for modeling neurodegenerative diseases affecting the corticospinal tract and enables therapeutic discovery efforts targeting corticospinal pathology.

RESULTS

Generation of regionally specific hiPSC-derived neuron and astrocyte populations

We sought to design a versatile, easily manipulable culture system incorporating region-specific cell lineages; neurons, and astrocytes. We differentiated healthy control hiPSCs into CNs, including a subset of long projecting corticospinal neurons (CSN), SpMNs, as well as cortical astrocytes (CAs) and spinal astrocytes (SpAs) (Figures 1A–1D). In order to maintain consistency across the experimental design, hiPSC-CNs as well as hiPSC-SpMNs were all derived from a single control hiPSC line (CS9XH7, Cedars Sinai). Healthy hiPSC CAs and SpAs, derived from a separate control hiPSC line (WC-30), and previously characterized for their regional identity (BX-0600 and BX-0650, respectively, BrainXell, Madison, WI, USA), were also incorporated (Figures 1B and 1D). While we chose to confirm the region-specific identity with the historically most relevant markers, further transcriptomic profiling and multi-marker characterization were done by Li et al. demonstrating clear telencephalic versus spinal regional identities.[32,33]

hiPSC-CNs were differentiated (Figures 1A and 1F) in high purity with 91.1% ± 0.7% immunostaining for the upper-layer CN marker, special AT-rich sequence-binding protein 2 (SATB2). A subset (27% ± 2.1%) of hiPSC-CNs also expressed chicken ovalbumin upstream promoter transcription factor (COUP-TF)-interacting protein 2 (CTIP2), a marker of subcerebral projection CSN lineage.[34]

Using methods to generate relatively pure SpMNs (Figures 1C and 1F),[35] our differentiation protocol yielded 96% ± 1.9%

(F) Quantification of neuronal markers shows that a large subset of TUJ1+ spinal neurons displays cholinergic spinal identity (ChAT+) as well as ISL1/2 expression, while cortical neuron differentiation protocol generates SATB2+ and CTIP2+ cortical neuron subtypes.

(G) Quantification of widely used astrocytic markers for both our astrocytic types to further identify them showing high levels of GFAP and S100β.

(H) Comparison of GFAP expression at two time points, DIV20 before plating with neurons (i.e., monocultures) and DIV48 (after 1 month of co-culture with cortical neurons and spinal motor neurons, respectively, in the chip), shows statistically significant increase of the GFAP+ levels in both cortical and spinal co-cultures. *t* test; *$p < 0.05$, **$p < 0.01$, ***$p < 0.001$, and ****$p < 0.0001$.

Scale bars, 40 μm. Data are represented as mean ± SEM

cholinergic motor neurons as defined by choline acetyltransferase (ChAT) immunostaining. A significant subset of these SpMNs also expressed the motor neuron transcription factor ISL1/2 (65% ± 8.6%). Confirming the region specificity of the protocol, fewer than 2% of SpMN cultures exhibited region-specific CN phenotypes.

Previous studies have utilized specific differentiation methods to generate hiPSC-derived CAs[36] and SpAs.[32,37] The schematic for astrocytic differentiation was a modified version of the BrainXell protocol[32] (Figures 1B and 1D). We utilized these regionally specific astrocytes to allow for an added layer of regional specificity and fidelity to the platform. hiPSC-derived CAs expressed markers of astrocyte lineage (50% ± 2.8% GFAP and 93.7% ± 1.7% S100β) as well as a marker of cortical identity (OTX2 92% ± 1.7%) (Figures 1B–1E and 1G). hiPSC-SpAs showed 57% ± 7.9% GFAP and 95% ± 1.4% S100β expression as well as a marker of spinal lineage HOXB4 (91% ± 2.5%). Fewer than 2% (1.6% ± 0.4%) of CAs expressed HOXB4, and only 5.5% ± 1.2% of SpA expressed OTX2 (Figure 1D, 1E, and 1G). Together, these results validate the establishment of region-specific neuronal and astrocytic subtypes modeling corticospinal connectivity.

We have previously demonstrated that co-culturing astrocytes and neurons promotes reciprocal maturation without altering their regional identities. In the present study, we sought to confirm this observation within the current experimental paradigm. Comparing the GFAP expression of both types of regional astrocytes in monoculture and after 1 month in co-culture with their respective neurons (neuron to astrocyte ratio 2:1) showed a significant increase in GFAP expression following co-culture with neurons (p = 0.0028 and p=0.0224 for the CAs and the SpAs, respectively) (Figure 1H). Our choice of a 2:1 neuron to astrocyte ratio optimizes maturation and synaptogenesis as well as sustains robust, analyzable network dynamics in both spinal and cortical platforms and is in line with the neuron:astrocyte ratio we have used previously to detail neuron and astrocyte interactions.[19,38–40] It is important to note that applying Ara-C at the neural progenitor cells (NPCs) during neuronal maturation is crucial to avoid the generation and proliferation of glial cells that can change the co-culture ratios in the chip.

Design of microfluidic device for corticospinal tract modeling

In order to provide spatial separation of the cortical and spinal compartments, we bioengineered a dual chamber PDMS (polydimethylsiloxane) microfluidic device that allows for imaging of cell subtypes and incorporates an MEA for electrophysiological study. Each chamber was calculated to have 3.69 cm^2 surface area, which allowed for a plating density of 6–8 $\times$ $10^4/cm^2$ cells per chamber. This density was optimized to ensure sufficient coverage of the electrodes while minimizing the total number of cells required. Neurite connectivity between the two chambers was accomplished by incorporating 100 microchannels. To minimize fluidic exchange but allow for cortical neurites to extend distally into the spinal chamber, the microchannels had a width of 8–10 μm and a length of 450 μm (Figure 2A). We specifically employed box-shaped PDMS microfluidic devices in our study due to several technical advantages. Their open-top design allows for homogeneous surface coating, ensuring consistent distribution of extracellular matrix proteins essential for cell adhesion and growth and also enables the direct addition of small molecules (as well as viral vectors) onto the culture surface, promoting uniform distribution across the entire cell population.

To encourage unidirectional cortical neurite growth from the cortical to spinal compartment, CNs were seeded first and their axons were guided through microchannels using media enriched with brain-derived neurotrophic factor and glial cell line-derived neurotrophic factor for chemoattraction to the spinal compartment. We observed that plating CNs alone for about 5–7 days facilitates axonal outgrowth and ensures that axons will be able to pass through the microchannels and will not be hindered by newly plated astrocytes whose small, rounded somas can occupy the channels before they adhere and flatten. This strategy ensures the unidirectional growth of cortical neuronal axons and discourages the retrograde growth of spinal neuron axons. Cortical neurons were transduced with CAMKII-ChR2-YFP upon plating, enabling visualization of axon growth through the microchannels and into the spinal compartment (Figure 2B). After 4–6 days, CAs, SpMNs, and SpAs were introduced to complete the cortical and spinal co-cultures into a corticospinal tract-like arrangement (Figures 2A and 2C). This protocol yielded two spatially distinct, microfluidically separated compartments containing CSNs and CAs in one and SpMNs and SpAs in the other. Significant axonal outgrowth from CNs was observed within the spinal compartment (Figure 2B). The absence of significant media exchange between the two chambers was temporally confirmed using a live cell dye (NucSpot Live 488). The live cell dye applied into the cortical compartment was taken up by CNs within 10 min of application, but there was no live cell staining in the spinal compartment for periods exceeding 24 h (Figures S1A and S1B).

To further confirm microfluidic separation, we evaluated whether there was significant migration of cells between the two chambers. For this purpose, cells were seeded in the cortical compartment and migration was assessed 1 week later, by counting Hoechst+ nuclei in the spinal compartment, which was initially devoid of seeded cells. The quantification of Hoechst+ nuclei in this compartment confirmed minimal migration through the microchannels. On average, we observed 8.4 ± 2 cells present in the spinal compartment per device (n = 6 devices) of the 22,500 cells (15,000 neurons and 7,500 astrocytes) seeded in the compartment, indicating that only a small number of cells migrated to the adjacent compartment (Figure 2D).

Finally, to assess long-term cell migration in co-culture, reflecting conditions used in our extended experimental paradigms, we performed immunostaining 4 weeks after plating and assessed the presence of cortical and spinal neuronal markers. In the cortical compartment, we immunostained for spinal markers HOXB4 (from TUJ1-, SpA) and ChAT (from TUJ1+, SpMN) (Figure 2E), while in the spinal compartment, we immunostained for cortical markers SATB2 (from TUJ1+, CN) and OTX2 (from TUJ1-, CA) (Figure 2F). Our analysis revealed that only 4.0% ± 1.0% of astrocytes in the spinal

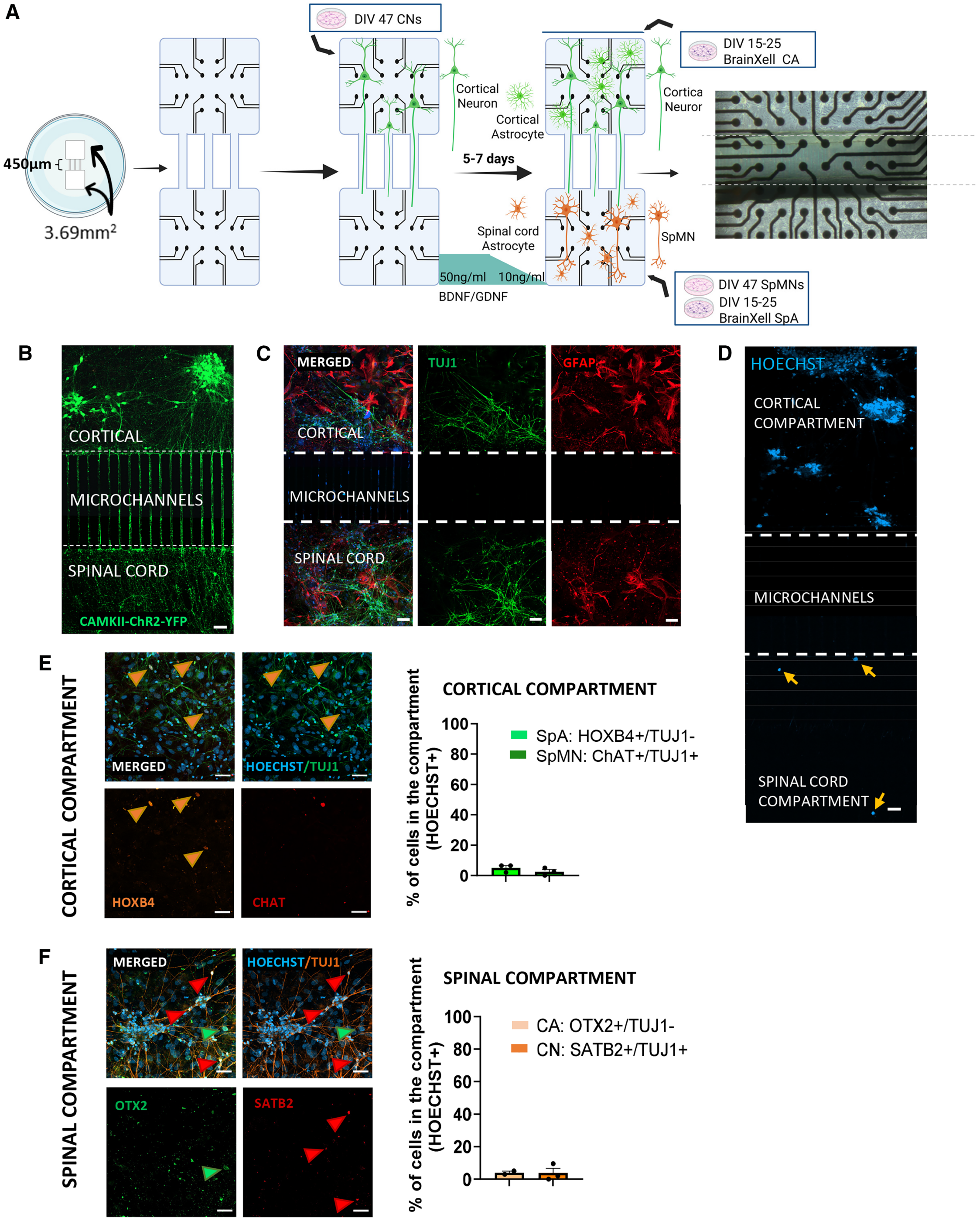

Figure 2. Dual microfluidic chamber design models corticospinal tract

(A) Schematic diagram demonstrating the culture of hiPSC-CNs followed by CAs, SpMNs, and SpAs on dual-chambered box microfluidic devices connected through microchannels and bonded on MEA plates.

(B) CAMKII-ChR2-YFP-transduced cortical neurons identify excitatory CNs and demonstrate neurite-abundant outgrowth from cortical to spinal compartment.

(C) Immunohistochemical staining identifies TUJ1+ neuron and GFAP+ astrocyte interactions in each of this dual-chambered co-culture platform.

(D) Nuclear staining (Hoechst) showing minimal migration of cells from one compartment to the other through the microchannels (8 ± 2 cells/device, 6 devices).

(legend continued on next page)

compartment were OTX2+ and only 3.8% ± 2.9% of neurons were SATB2+ (Figure 2F), suggesting their migration from the cortical compartment. Conversely, 5.0% ± 1.5% of astrocytes in the cortical compartment were HOXB4+ and 2.5% ± 1.4% of neurons were ChAT+, suggestive of a spinal origin (Figure 2E). These results indicate very little cellular migration between the two chambers, representing only a small fraction of the total seeded cells per compartment, and highlight that the vast majority of cells remain within their designated compartments, even after prolonged culture time. It is worth noting that a small percentage of the neurons and astrocytes in monoculture (Figures 1E and 1F) also exhibit low levels of markers not typically associated with their expected lineage ("opposite" markers), suggesting that actual migration might be even more limited.

Recapitulating the human corticospinal tract: Region-specific marker expression in hiPSC-derived cultures

To confirm that the regional identity of cell subtypes was maintained following co-culture in this microfluidic platform, we examined relevant cell subtype markers of neuronal and glial identity. After 1 month in co-culture, immunostaining confirmed the preservation of region-specific marker expression in the compartmentalized system, closely mirroring the observations in monocultures. In the cortical compartment (Figure 3A), 98.6% ± 0.6% of neurons expressed the upper layer neuron marker SATB2, while 27.2% ± 1.7% also expressed CTIP2, consistent with layer V CSN identity. Most CNs were excitatory (CAMKII+ 93.6% ± 0.9%), with a smaller population of inhibitory neurons (GAD67+ 15.9% ± 1.8%). CAs retained their region-specific identity, with 94.3% ± 0.7% expressing OTX2 and 72.5% ± 5.8% expressing GFAP, indicating preserved maturation in co-culture. In the spinal compartment (Figure 3B), neurons expressed 95.9% ± 0.7% ChAT and 87.5% ± 1.3% ISL1/2 along with 6.4% ± 1.1% GAD67, confirming their motor neuron identity and the presence of small subset of inhibitory interneurons, respectively. SpAs showed 86.0% ± 3.1% GFAP immunostaining and 91.5% ± 0.9% HOXB4 immunostaining, supporting their astrocytic and regional identities.

To evaluate motor neuron maturation in the CST platform, we characterized neurite complexity in both the cortical and spinal compartments using Sholl analysis (ImageJ software). At 10 weeks in culture, both CNs and SpMNs exhibited increased neurite complexity compared to an earlier time point (week 1), as evidenced by a greater number of neurite intersections across all radii (Figures 3C and 3D). Cortical neurons showed a peak number of intersections at a radius of 60 μm, while SpMNs peaked at 40 μm, indicating that, over time, CNs develop more extended and elaborate arborization distally from the soma (Figures 3C and 3D).

These data, taken together, demonstrate that the corticospinal tract-on-a-chip maintains region-specific cellular identities and suggest that the morphological neuronal maturation we observed in the co-cultures was not due to shifts in regional identity but was an expression of emerging properties of co-cultures and their network connections through the corticospinal tract (Figures 3A–3D).

Electrophysiological properties and maturation of the corticospinal tract-on-a-chip

To evaluate electrophysiological maturation and emerging network activity across the corticospinal tract and within each compartment, we performed MEA voltage recordings at weekly intervals over a 12-week period. Example raster plots of spike counts per electrode demonstrated a progressive increase in activity over time (Figure 4A). Quantitative analysis of weighted mean firing rate (wMFR), number of active electrodes, burst percentage, average burst frequency, burst duration, and network burst frequency revealed a consistent increase over time. For the analysis, all electrophysiological metrics were computed at the well level (whole chip), pooling electrodes from both the cortical and spinal compartments to capture device-wide maturation, while excluding the electrodes on top of microchannels. Direct comparison between an early (week 1) and a late (week 12) time point showed statistically significant increases across all parameters ($p = 0.0044$, $p = 0.0001$, $p = 0.0002$, $p < 0.0001$, $p = 0.0002$ respectively) except network burst frequency that reached significancy when comparing to week 11 ($p = 0.0303$) but not between weeks 1 and 12. Synchrony parameters, such as the synchrony index and area under normalized cross-correlation (AUNCC), similarly demonstrated significant increases (week1 versus week12, $p = 0.0036$ and $p = 0.0003$ respectively) indicating enhanced network activity over the time course of the study (Figure 4B). All measured electrophysiological parameters plateaued between weeks 10 and 12, suggesting the network reached a stable state of electrophysiological maturation. Astrocytes plated in the chips without the neurons did not show any electrophysiological activity (Figure 4B).

We were also interested in investigating the electrophysiological maturation pattern in our model in the absence of astrocytes. We plated the CNs alone in one compartment and 5–7 days later, introduced the spinal neurons in the other compartment. Weekly recordings were performed and compared to measurements from the complete co-culture setup with neurons and astrocytes (Figure 4B). Expectedly, neuron-only chips showed substantially fewer active electrodes ($p < 0.0001$) as neuronal populations tend to cluster without the support of the glial component. The rest of the electrophysiological parameters studied reached a much earlier plateau at around 4–5 weeks of culture in the chip and remained markedly lower compared to the co-culture of neurons and astrocytes over the 12-week study (wMFR $p = 0.0123$, burst duration $p = 0.0411$, burst frequency $p = 0.136$, burst percentage $p = 0.0051$). Network activity parameters were also statistically

(E and F) Quantification of cells migrating (E) from the spinal to the cortical (orange arrows are indicative of SpA: HOXB4+TUJ1−; no ChAT+ SpMNs are present) and (F) from the cortical to the spinal compartment (green arrows indicate CA: OTX2+TUJ1−, and red arrows indicate CN: SATB2+TUJ1+), using region-specific immunohistochemical markers 1 month after plating. Graphs show the percentage of cells that expressed an opposite regional identity marker relative to the total counted cells (at least 250 Hoechst+TUJ+ neurons and at least 250 Hoechst+TUJ− astrocytes for each compartment).
Scale bars, 50 μm. Data are represented as mean ± SEM.

CellPress
OPEN ACCESS

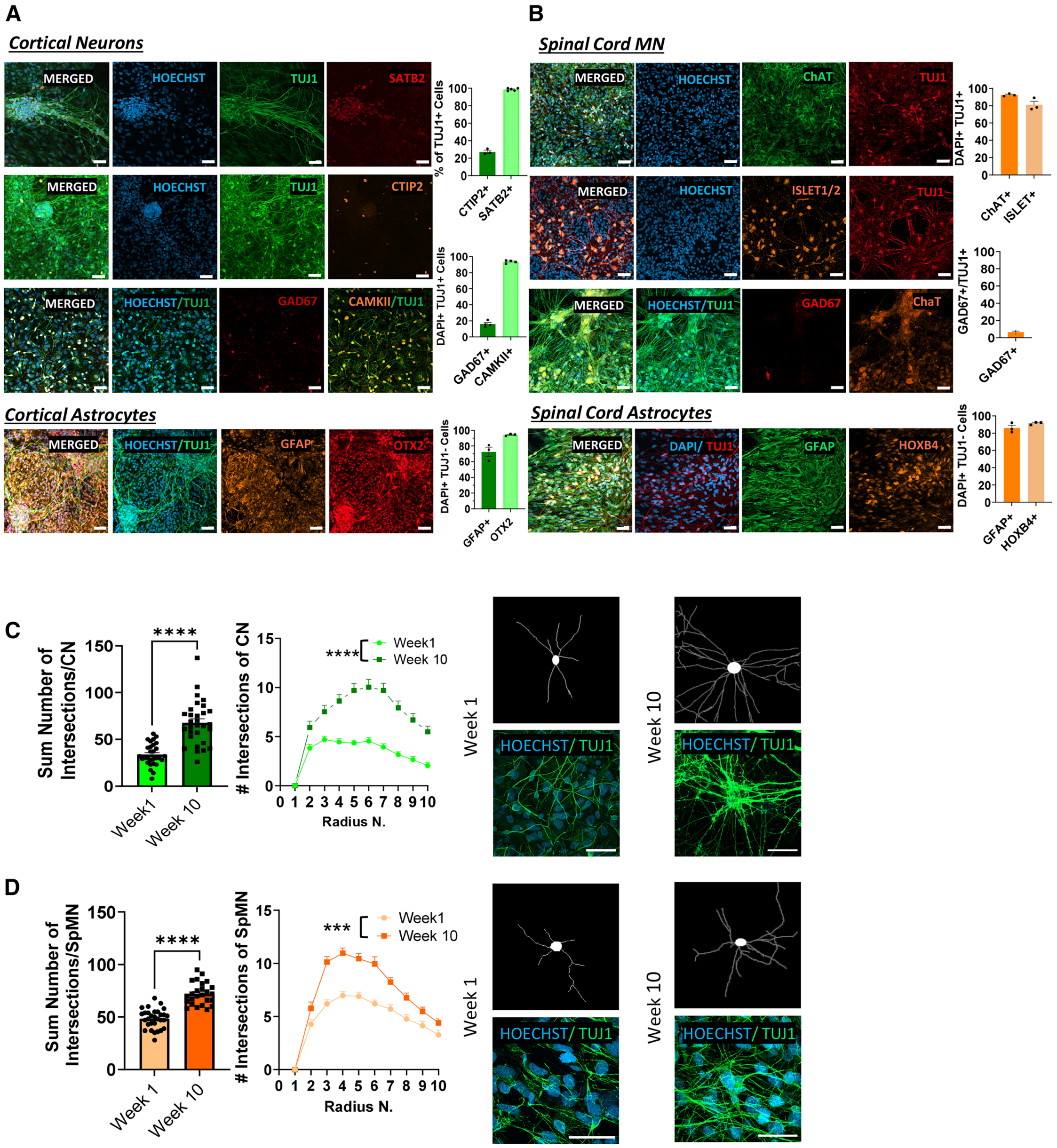

Figure 3. Co-culture of regional-specific neurons and astrocytes recapitulates cortical and spinal cellular maturation

(A) Following the co-culture of CNs with CAs, regional cell subtype identity is maintained with >95% SATB2 identification and 26% CTIP2+ subcerebral projection neurons. A minority of CNs expressed GAD67 indicative of inhibitory interneuron differentiation. Cortical astrocytes' staining shows the majority of CAs expressing GFAP and nearly all expressing the cortical-regional identity marker OTX2.

(B) Highly efficient SpMN differentiation was supported by elevated ChAT and ISL1/2 expression. A minority of spinal neurons expressed the inhibitory interneuron marker GAD67. Spinal astrocytes also showed high expression of GFAP and spinal-regional identity marker HOXB4. Scale bars, 40 μm.

(C and D) Sholl analysis of neurite outgrowth in CN and SpMN co-cultures at 1 and 10 weeks *in vitro* ($n = 30$ neurons per condition) with representative source and analysis images. One-way ANOVA and t test, $***p < 0.001$ and $****p < 0.0001$.

Scale bars, 50 μm. Data are represented as mean ± SEM.

significantly lower over time (network burst frequency $p = 0.0271$, synchrony index $p = 0.0041$, AUNCC $p = 0.0108$). These results demonstrate that astrocytes influence dynamic functional changes in corticospinal tract maturation.

In addition, we investigated differences in network maturation between corticospinal chips (CNs and astrocytes on one side and spinal neurons and astrocytes to the other), compared to single-population chips containing only a single region-specific co-culture (spinal co-cultures of neurons and astrocytes or cortical co-cultures of neurons and astrocytes on one side of the "single-population chips") (schematic of the comparison in Figures S2A and S2B). MEA

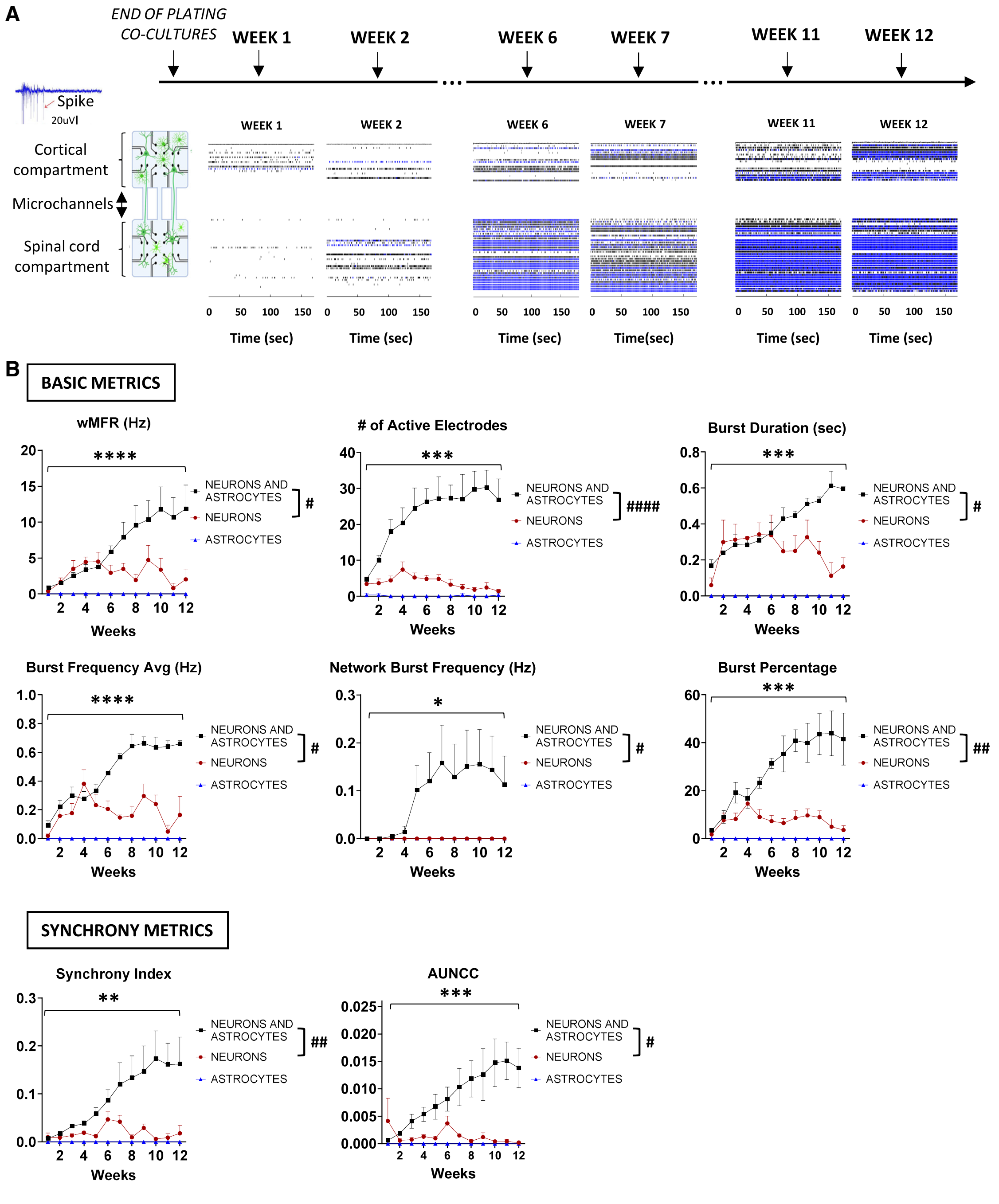

Figure 4. Longitudinal recordings reveal electrophysiological maturation of region-specific neuronal activity

(A) Raster plots of longitudinal multielectrode array (MEA) recordings show increases of spontaneous activity in both the cortical (upper) and spinal (lower) compartments over 12 weeks in co-culture (microchannels between cortical and spinal compartments excluded from analysis).

(B) Quantification of electrophysiological parameters measured from combining the electrodes from both compartments of each CST-on-a-chip shows increases in all basic metrics with plateaus in activity after 8–10 weeks (black curves, $n = 14$ devices, 3 biological replicates). Comparisons in asterisk (one-way ANOVA) refer to week 1 versus week 12. In addition, quantification of electrophysiological parameters of neuron-only cultures is shown in red curves and astrocyte-only cultures in blue curves. Graphs show early plateau of all metrics in neuron-only cultures after 2–4 weeks. Comparison of electrophysiological activity between neuron-only and neurons with astrocytes reveals statistically significant higher values in the co-culture condition for all basic metrics over time ($n = 3$–5 replicates per group). Comparisons in pound (two-way ANOVA) are made over the time course of 12 weeks. Astrocytes, as expected, show no activity in all electrophysiological metrics. $^{\#,*}p < 0.05$, $^{\#\#,**}p < 0.01$, $^{***}p < 0.001$, and $^{\#\#\#\#,****}p < 0.0001$. wMFR, weighted mean firing rate; and AUNCC, area under normalized cross-correlation. Data are represented as mean ± SEM.

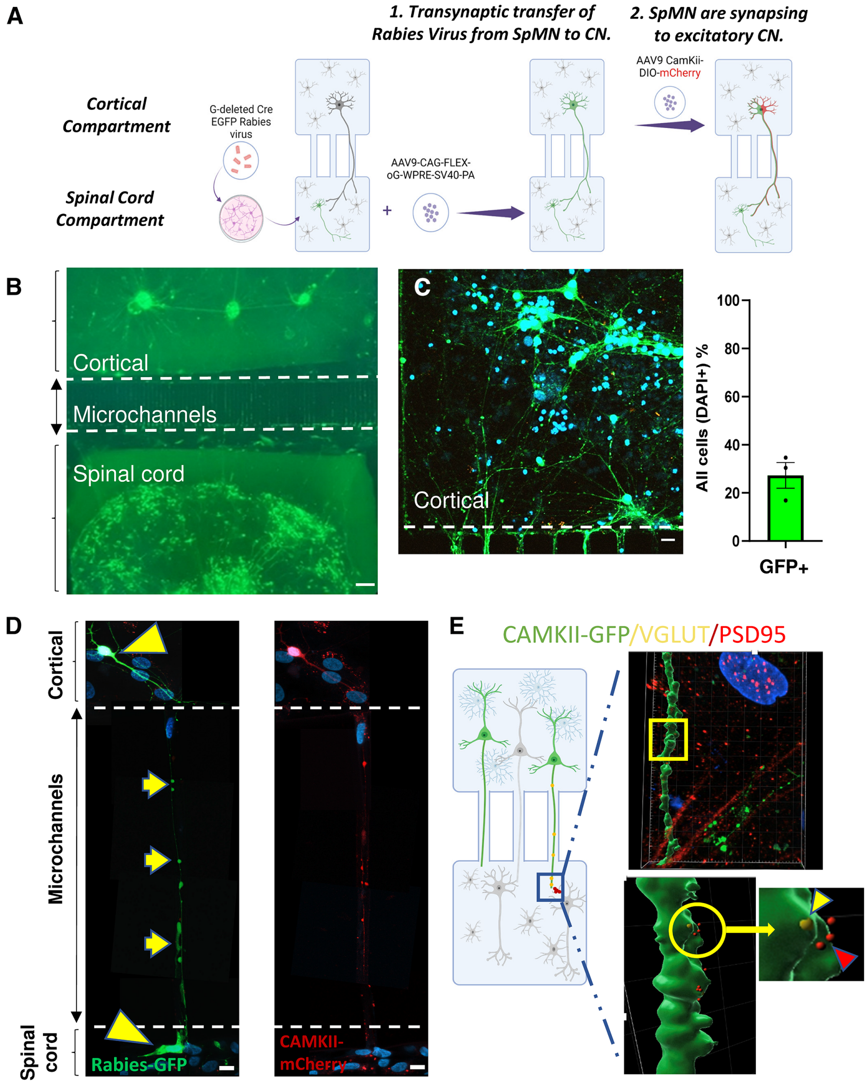

Figure 5. Morphologic characterization of corticospinal and spinal motor neuron connectivity

(A) Schematic diagram demonstrating administration of G-deleted Cre-EGFP rabies virus to SpMNs. This was followed a week later by application of the helper virus AAV-CAG-FLEX-oG-WPRE-SV40-PA to SpMNs. AAV9 CAMKII-DIO-mCherry was added to the cortical compartment to transduce and selectively distinguish the excitatory CNs co-infected with rabies.

(B) GFP+ neurons were noted in both the spinal and subsequently in the cortical compartments indicating trans-synaptic viral transport from SpMNs to CNs. Scale bar, 100 μm.

(C) Quantification of GFP+ cortical neurons 1 week after G-deleted Cre-EGFP rabies virus infection of SpMNs reveals that 27.26% ± 5.34% CNs were synaptically connected with SpMNs. Scale bar, 20 μm.

(D) mCherry and GFP co-localization demonstrates the connectivity of excitatory CN subpopulations with spinal motor neurons. Arrows indicate neuron cell soma Scale bars, 10 μm.

(E) Immunostaining and subsequent Imaris 3D processing of CAMKII-GFP+ CN neurites in the spinal compartment shows co-localization (within 1 μm from the axonal volume) of presynaptic (VGLUT+, minimum diameter of 0.5 μm) glutamatergic marker from CN with the postsynaptic (PSD95+, minimum diameter of 0.3 μm) marker from SpMN.

See also Video S1.

Data are represented as mean ± SEM.

recordings were obtained weekly over a 12-week period to assess electrophysiologic network maturation (Figures S2A and S2B).

Culturing of hiPSC-CNs in cortical compartment with hiPSC-SpMNs in spinal compartment of "corticospinal chips" compared to CNs in a single-population chip improved the average burst frequency ($p = 0.0169$) as well as the burst percentage ($p = 0.045$) across the time of the study (Figure S2A). Further, these hiPSC-CNs plated in corticospinal chips tended toward lower synchrony across the 12-week duration compared to CNs cultured in single-population chips. Conversely, culture conditions in the chip did not impact spinal neuronal maturation over a 12-week time course as seen across basic and synchrony metrics (Figure S2B). These findings suggest that the presence of both cortical and spinal neuronal populations, along with their respective astrocytes, may enhance network complexity and contribute to more robust and coordinated electrophysiological activity compared to single-population systems.

Structural evidence of synaptic connectivity between hiPSC-derived cortical and the spinal motor neuron populations

To enable our platform to model the corticospinal tract, a critical requirement was that it supports the formation of functional synaptic connections between CN and SpMN. To confirm synaptic connectivity, we employed a rabies virus trans-synaptic tracing strategy (Figure 5A). hiPSC-derived SpMNs were infected with a G-deleted Cre-EGFP rabies virus.[41] One week later, the helper virus AAV-CAG-FLEX-oG-WPRE-SV40-PA was introduced, enabling retrograde trans-synaptic transfer of rabies-GFP. Cortical neurons labeled with rabies-GFP (Figures 5B and 5C) confirmed that SpMN established trans-synaptic connections with CNs. To assess rabies-mediated retrograde transport in neurons, CNs were seeded in the cortical compartment and SpMNs in the spinal compartment, without the inclusion of their respective astrocytes. Using fluorescence microscopy to estimate the proportion of CNs connected to spinal neurons within the microfluidic device, we quantified rabies-GFP+ CNs following trans-synaptic labeling from Hoechst+ cells (CNs). This analysis revealed that 27% ± 5.3% of CNs were rabies-GFP+ and suggests that only a subset of CNs contributed to the corticospinal tract (Figure 5C). In parallel, we transduced

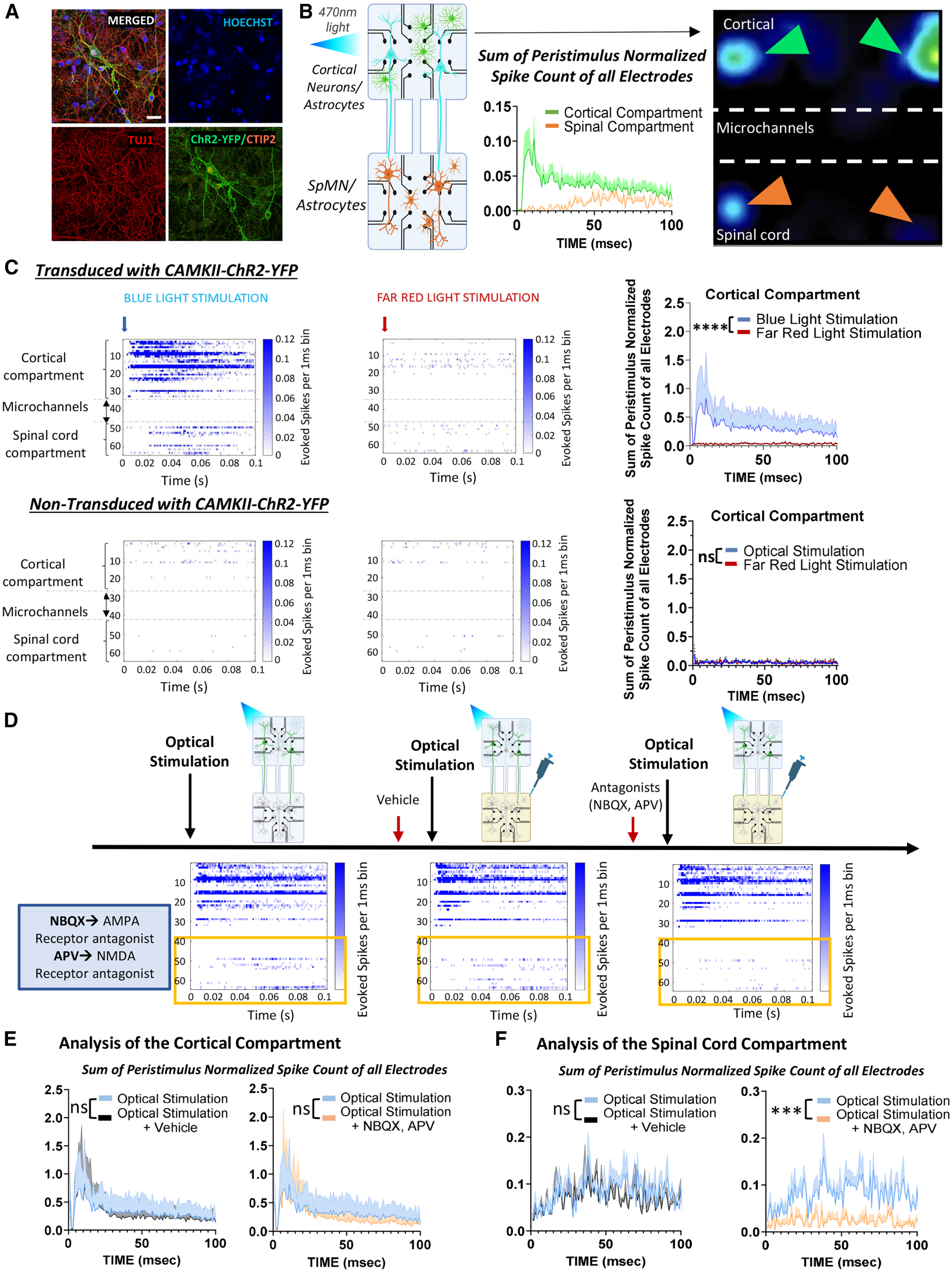

Figure 6. Optogenetic stimulation demonstrates network connectivity between excitatory CNs and SpMNs

(A) CAMKII-ChR2-YFP+ transduced in CNs co-localizes with subcerebral CTIP2+ in subcerebral projection CSNs. Scale bar, 20 μm.

(B) Optic stimulation of CAMKII-ChR2-YFP+ CN induces synchronized spike activity in MEA electrodes. Following optic stimulation, cortical neurons show summation of electrophysiological activity (green). This is temporally followed by electrophysiological responses from SpMN in the spinal compartment (orange). Visual heatmap representation demonstrates this synchronized activity across the CST.

(legend continued on next page)

the CNs with AAV9 CAMKII-DIO-mCherry. This construct was designed to express mCherry under the CAMKII promoter and in the presence of Cre recombinase, which was provided by the rabies GΔ Cre-EGFP virus.[42] Neurons that were double-positive (GFP+ and mCherry+) were identified as synaptically connected excitatory CNs, confirming corticospinal connectivity in this model (Figure 5D).

To further evaluate this synaptic connectivity morphologically, CNs were transduced with CAMKII-ChR2-YFP AAV9, enabling visualization of their cell bodies and axons via YFP expression. We utilized Imaris software (Oxford Instruments, Oxon, UK) to generate 3D reconstructions of YFP-positive axons and qualitatively assess the spatial relationships between synaptic markers. In the spinal compartment, immunostaining with anti-YFP identified cortical axons, while antibodies against the presynaptic vesicular glutamate transporter (VGLUT) and the postsynaptic marker postsynaptic density 95 (PSD95) labeled synaptic elements. VGLUT-positive puncta (with a minimum diameter of 0.5 μm) located within the surface of YFP-positive axons were considered indicative of presynaptic puncta. PSD95-positive puncta (with a minimum diameter of 0.3 μm) located within 1 μm from the axonal volume were interpreted as postsynaptic sites.[40] The observed spatial proximity of VGLUT and PSD95 puncta in 3D reconstructions supported the presence of synaptic connectivity between CNs and SpMNs (Figure 5E, Video S1). Together, these findings provide structural evidence of synaptic connections between CNs and SpMNs within the platform, recapitulating the *in vivo* organization of long-range corticospinal projections in which CNs form synapses with SpMNs.

Optical stimulation reveals functional evidence of synaptic connectivity in the corticospinal tract-on-a-chip

Functional connectivity between cortical and spinal neurons was examined using MEA recordings and optogenetic stimulation of CNs transduced with CAMKII-ChR2-YFP. Immunostaining of transduced neurons confirmed co-localization of CAMKII-ChR2-YFP with the subcerebral projection neuron marker CTIP2 and the pan-neuronal marker TUJ1, indicating the expression of ChR2 in excitatory CSNs (Figure 6A). Corticospinal tract-on-a-chip cultures were matured on MEA plates and stimulated with blue light (470 nm) while recording the response from cortical and spinal compartments to determine light-evoked neuronal activity and synaptic transmission through the corticospinal network. Peristimulus spike count normalized to baseline activity demonstrated an immediate increase in spiking in the cortical compartment, followed by a delayed response in the spinal compartment, indicative of a temporal lag from axonal and synaptic transmission (Figure 6B).

To confirm that the observed neuronal response was specifically mediated by ChR2-YFP activation, we repeated the experiment comparing blue to far-red light stimulation (Figure 6C). While there was robust activation of CNs in response to blue light, far-red light elicited no significant response ($p < 0.0001$) (Figure 6C). To further validate the specificity of ChR2-YFP-mediated activation, blue light stimulation of non-transduced hiPSC-CNs (i.e., CNs not expressing ChR2-YFP) did not elicit any electrophysiological response, with no significant difference compared to far-red light stimulation ($p = 0.9493$; Figure 6C). Peristimulus spike count showed that blue light stimulation of CNs induced a distal response in SpMNs (Figure 6F). These findings demonstrate that optical stimulation of CNs induces a synaptic response in SpMNs, suggesting functional connectivity between the two neuronal populations in this platform through the corticospinal tract axons.

By contrast, there is no reverse (spinal to cortical) functional connectivity in our model. To assess this, we transduced the spinal compartment with AAV9-Syn-ChrimsonR-tdT and confirmed opsin expression in spinal neurons under the synapsin promoter (active only in neuronal populations) by tdTomato fluorescence imaging (Figure S3A). When we flashed 590-nm (orange) light at the chip, we proved reliable excitation of the spinal neurons specific to the Chrimson opsin (no response at non-specific green light stimulations) (Figure S3B). Most importantly, we show no downstream excitation of the CNs indicating minimal, if any, presence of spinal neuronal axons at the cortical compartment and further supporting the unidirectional growth of the model (Figure S3C).

To investigate whether the SpMN response to cortical optical stimulation was mediated by the neurotransmitter glutamate, we utilized a pharmacological approach and considered three conditions: (1) blue light stimulation under baseline conditions, (2) blue light stimulation following the addition of vehicle solution, and (3) blue light stimulation following the addition of a cocktail containing the AMPA receptor antagonist 2,3-dihydroxy-6-nitro-7-sulfamoyl-benzo[f]quinoxaline (NBQX) and the NMDA receptor antagonist 2-amino-5-phosphonovaleric acid (APV) (Figure 6D). Raster plots showed a consistent electrophysiological response from hiPSC-SpMNs following the optical activation of CAMKII-ChR2-YFP+ hiPSC-CNs under baseline and vehicle conditions ($n = 4$ biological replicates). Quantification revealed no statistical difference in either compartment in the peristimulus spike count before and after the addition of vehicle (Figure 6E). However, blocking AMPA and NMDA receptors with NBQX and APV led to a significant decrease ($p=0.0009$) in spinal

(C) Peristimulus raster plots (1-ms bin) of CAMKII-ChR2-YFP+-transduced CN show activity in the cortical compartment followed by an electrophysiological response in the spinal compartment (top, left). Far-red light stimulation does not activate these CNs (top, right). Similarly, neither blue light nor far-red light stimulation activates non-transduced CNs (bottom). Peristimulus plots are extracted from 5-min blue light and far-red light stimulation experiments (70 light pulses) of 10-week co-cultures ($N = 3$). Wilcoxon matched-pairs signed rank test. ****$p < 0.0001$. Error bars ±SEM are presented as shaded blue area.

(D) Peristimulus raster plots (1-ms bin) recorded following the addition of the AMPA receptor antagonist NBQX and NMDA receptor antagonist APV to the spinal compartment shows the reduction in electrophysiological activity in the spinal compartment with sparing of activity in the cortical compartment.

(E) Peristimulus electrophysiological activity in the cortical compartment remains unaffected regardless of the treatment conditions ($n = 4$).

(F) Glutamatergic activity is reduced in the spinal compartment following application of glutamate antagonists ($n = 4$). Stimulation parameters: 70 stimuli, 0.25 Hz, 5-ms stimulus duration, 30% intensity. Error bars are represented as shaded area and indicate mean ± SEM. One-way ANOVA, ***$p < 0.001$.

compartment activity (Figure 6F), while hiPSC-CN activity remained unaffected (Figure 6E). These findings confirm that the spinal neuronal response to cortical optical stimulation is mediated by glutamatergic transmission, providing further evidence of functional synaptic connectivity within the corticospinal tract-on-a-chip.

DISCUSSION

Modeling corticospinal connectivity in healthy control conditions and in neurological disorders presents a distinct challenge due to the inherent structural and functional complexity of this long-range projection pathway. Traditionally, studies of the corticospinal tract and its synaptic connectivity have relied heavily on animal models[7] with notable limitations in capturing the cellular and molecular mechanisms underlying human pathobiology.[43,44] Non-human primate models may provide a closer approximation to human neuroanatomy and disease mechanisms but are also limited in their ability to recapitulate neurodegenerative disorders that involve complex or unidentified genetic and environmental etiologies, such as PPMS and sporadic ALS. In addition, their use is constrained by significant ethical, logistical, and financial challenges.[45,46] The development of a humanized corticospinal tract-on-a-chip is our innovative approach to address some of these challenges.

hiPSC-derived neuronal cultures are emerging methods for examining a host of intrinsic cell properties in both health and in neurodegenerative disease.[21,47–51] Donor-derived iPSCs enable the generation of disease-relevant neuronal subtypes, including corticospinal neurons and SpMNs, which have the potential to recapitulate key pathological features of disorders with prominent corticospinal pathology, including ALS and HSP, such as TDP-43 mislocalization and axonal degeneration.[21,23] For this platform, we employed optimized protocols to generate highly pure populations of CNs, SpMNs and already highly characterized region-specific astrocytes from hiPSCs.[32,33] Our findings notably demonstrate that the iPSC-derived cell subtypes acquire molecular signatures characteristic of their *in vivo* counterparts. Approximately one-fourth of the CNs exhibited characteristic layer V markers such as CTIP2 that have been described *in vivo* as indicative of subcerebral projection neurons shown to degenerate in models of neurodegeneration.[10] Moreover, spinal neurons expressed ChAT and ISL1/2 in high abundancy, while astrocytes were regionally patterned to express forebrain (OTX2) or spinal (HOXB4) transcription factors, as astrocyte heterogeneity is recognized as important to both normal biology and pathology.[52–54] *In vivo*, astrocytes from different brain regions such as the cortex, hippocampus, midbrain, and cerebellum exhibit distinct synaptogenic profiles driven by differences in the expression of synaptogenic factors.[55,56]

One of the goals of this study was to engineer a chip with a more physiologically relevant microenvironment. With these four fully characterized cell types, we aimed to bridge this gap combining these populations in an integrated manner, while most studies using hiPSC-derived CSNs and SpMNs have utilized monocultures or simple co-cultures with glial cells. It is well established that co-culturing neurons with astrocytes enhances synaptogenesis, with astrocyte-conditioned media alone sufficient to increase synaptic density.[57] Additionally, direct neuron-astrocyte interactions influence astrocyte gene expression[58] as also evidenced by increased GFAP expression in astrocytes co-cultured with neurons compared to monocultures.[19] In this study, we showed that co-culturing neurons and astrocytes in our system also resulted in increased GFAP expression compared to astrocyte monocultures, further supporting the role of neuronal interactions in astrocyte maturation, and vice versa.[59,60] Future studies could incorporate additional cell types, such as oligodendrocytes and microglia, to further enhance the physiological relevance of the model. This expansion may provide deeper insights into cell-type-specific contributions and interactions within the corticospinal circuit, building upon the foundational framework established with the current two-cell-type system.

To combine the cell types in a single design, we leveraged recent advances in bioengineering technologies that have expanded the capabilities of iPSC-based platforms. Some platforms enable the generation of 3D brain and spinal cord organoids that self-organize to mimic aspects of human neural development, including cortical-cortical and motor unit connectivity, and synapse formation.[24,61] Building on these developments, the integration of microfluidic devices and scaffold-based assembloids has further enhanced the ability to interrogate synaptic biology, axon guidance, and long-range connectivity in a controlled and modular fashion.[24,30] These platforms have been used to model long-range projection systems, including visual, somatosensory, and motor pathways.[42,62] Considering both the strengths and the limitations of these 3D systems (e.g., reduced experimental control over individual cell types and media conditions, limited spatial and temporal resolution, and challenges in quantitatively assessing functionally and morphologically relevant outcomes), we underscore the critical need for refined platforms that combine anatomical fidelity with experimental versatility to effectively model corticospinal connectivity.

To address these requirements, we developed a hiPSC-based *in vitro* model of the corticospinal tract that integrates the scalability and accessibility of 2D cultures with the spatial resolution afforded by microfluidic compartmentalization. Sophisticated microfluidic designs, often referred to as "lab-on-a-chip" systems, have paved the way for innovative approaches to studying the pathophysiology of neurodegenerative diseases using these region-specific iPSCs.[63,64] We chose an open-top-style compartmentalized box device that required small numbers of cells, as well as allowed for long-term survival and homogeneous distribution of neurons that was important for the longitudinal assessment of neuronal morphology and electrophysiology.

To further replicate corticospinal tract morphology, an emphasis on unidirectional neurite outgrowth was planned. The plating of corticospinal neurons prior to SpMN as well as the encouragement of unilateral growth of neurites with the addition of growth factors helped to ensure this occurred. Filling the narrow width of the microchannels with corticospinal neurites also discouraged retrograde growth and significant cell migration across the compartments.

Importantly, we show that CNs plated in this dual-chambered microfluidic system retain high cortical identity without acquiring

spinal signatures. Similarly, SpMNs maintained high purity in both monoculture and dual-chamber formats, confirmed by ChAT and ISL1/2 expression. We propose that this platform will be useful for modeling neurodegenerative diseases involving axonal transport deficits, synaptic dysfunction, and neuron-glia interactions, areas that are difficult to investigate using traditional culture formats.[24,30]

Our system further incorporates MEA technology to enable longitudinal assessment of electrophysiological maturation and network activity of the hiPSC-derived corticospinal tract over time. By ensuring coverage of both compartments with electrodes, the platform supports simultaneous electrophysiological measurements from cortical and spinal chambers. Our data demonstrate direct measurement of neural activity at both compartments as well as progressive increases in spontaneous activity and corticospinal synchrony over time, with an electrophysiological plateau observed around 8–10 weeks of co-culture.

We and others have previously described MEA-based physiological maturation of CNs and SpMNs in the presence or absence of astrocytes.[19,40] Studies comparing neuronal cultures originating from the prefrontal cortex, hippocampus, amygdala, and spinal cord have shown unique electrophysiological patterns for each subtype. Specifically, changes in spike rate, interspike interval, time between bursts, and burst frequency were found to be region specific for hiPSC-derived neurons. While Dauth et al. reported minimal differences in electrophysiological metrics like firing rate and interburst interval across neuronal subtypes within a multiregional brain-on-a-chip model, they observed clear differences when these populations were compared to their respective monocultures.[65] This indicates that the functional interaction between regionally distinct neuronal types alters their maturation dynamics and shapes the emergent electrophysiological properties of the network.[66] In our analysis, we observed a trend toward lower levels of synchrony in cortical co-cultures from the corticospinal chips, compared to CNs from the single-population chips. However, we did not find a similar effect on the electrophysiological maturation of SpMNs from the corticospinal chips compared to the single-population chips that had similar electrophysiological pattern of maturation in the parameters examined. It is worth noting that synchrony of SpMNs in the corticospinal chips had a trend toward lower levels compared to the single-population chips, showing a firing pattern closer to human physiology. These results support the idea that corticospinal interactions influence electrophysiological maturation.

In vivo, the neuron:astrocyte ratio varies by CNS region, approximating 1:1 in the cortex and 1:4 in the spinal cord ventral horn, with additional region-specific variability within the motor cortex and across spinal cord levels.[67,68] These ratios cannot be fully translated to *in vitro* astrocyte-neuron co-culture systems, as the *in vivo* CNS microenvironment includes additional glial cell types and a complex extracellular matrix that help maintain appropriate neuronal proximity and guide connectivity. For *in vitro* platforms, a 2:1 neuron:astrocyte seeding ratio has been widely adopted in MEA-based platforms. At this relative density, neurons are diffusely and uniformly distributed and region-specific astrocytes accelerate neuronal maturation and synaptogenesis while supporting stable, reproducible, and robust network activity, as demonstrated in both spinal[19,38] and cortical co-culture[39,40] experimental platforms.

Our MEA data show that independent and simultaneous recordings from either the cortical or spinal compartments were possible, allowing for studies that could examine the interplay between CNs and SpMNs. In addition, pharmacological blockade experiments proved that the selective inhibition of synaptic activity following the application of glutamate antagonists to the spinal compartment did not affect the cortical compartment, which also suggests that there is fluidic isolation of these compartments and the platform affords the ability to study these populations in isolation.

Electrophysiological studies have increased the appreciation for demonstrating neuronal maturation, particularly when using *in vitro* iPSC cultures.[69] Our findings highlight the importance of longitudinal time-course analysis *in vitro* in order to develop a more accurate representation of *in vivo* biology. The maturation and the stabilization of electrophysiological activity may be particularly relevant in the modeling of neurodegenerative disorders and should be taken into consideration in studies using humanized corticospinal tracts from iPSCs to investigate either healthy or disease states.

One more critical feature of this model is the ability to re-create appropriate corticospinal synaptic structure, synaptic receptor subtype, and synaptic transmission. In the literature, limited attention has been given to modeling the functional and structural connectivity between these neuronal populations in the context of a more complex network. To make an effective and relevant model, we wanted to demonstrate efficient connectivity between CNs and SpMNs. One approach to do this necessitated a retrograde tracing strategy with rabies virus, using a modified method described before for retrograde transport and synaptic connectivity in human-derived aggregoids. The use of AAV9 CAMKII-DIO-mCherry transduction in CNs also demonstrated that this connectivity was relevant to excitatory CNs.[36] A second confirmation was investigated using immunohistochemistry. We demonstrate that hiPSC-CNs, identified by CAMKII-ChR2-YFP, express the vesicular glutamate transporter VGLUT localized to neurites within the cortical compartment as well as in distal neurites extending, through microchannels, into the spinal compartment. Importantly, the presence of VGLUT at these distal neurites was associated with synapses to cholinergic SpMN as defined by postsynaptic PSD95 immunostaining.

To firmly establish that the morphological connectivity we described was physiologically and functionally relevant, we utilized a CAMKIIα-ChR2-YFP construct to selectively transduce excitatory CNs, enabling blue light-mediated activation of channelrhodopsin. This approach allowed us to synchronize CN depolarization and assess corresponding responses in SpMNs. This corticospinal activity appears to be mediated by glutamatergic excitatory neurotransmission since blocking of AMPA and NMDA receptors resulted in a suppression of the electrophysiological response in SpMNs.

Taken together, our data provide a foundation for a humanized corticospinal tract-on-a-chip that enables the integration of healthy and disease-relevant cell populations to interrogate cell-autonomous and non-cell-autonomous mechanisms of

corticospinal pathology, mechanisms of network disease propagation, while also permitting high-resolution assessment of network- and synapse-level disease phenotypes such as hyper- and hypoexcitability and altered corticospinal synaptic function.[70] This platform also provides a scalable framework for drug discovery strategies, enabling the evaluation of interventions targeting region-specific (cortical and/or spinal) neural populations, as well as their synaptic connections.

Limitations of the study

This work has some limitations. While the platform can support longer durations in culture, our maturation window was 12 weeks. However, several aspects of the corticospinal neuronal and network development (e.g., electrophysiological properties, synaptic refinement, and astrocyte-neuron interactions) likely continue to evolve beyond this time point. As such, the data presented here probably reflect an intermediate stage of maturation, and longer longitudinal experiments may uncover late-emerging network phenotypes. Our electrophysiology readouts are robust for longitudinal MEA-based population profiling to track network dynamics but do not reveal single-cell electrophysiological properties. Complementary patch-clamp or high-resolution optophysiology, especially at later time points, would add mechanistic depth. We also centered our analyses on excitatory cortical projections to SpMNs and did not systematically examine inhibitory interneuron contributions or quantify excitatory-inhibitory balance across compartments. Given the established role of inhibition in sculpting network synchrony and maturation, this remains an important variable. Finally, neurons were derived from a single control hiPSC line and astrocytes from vendor-specified sources, which helps control variability but may limit generalizability across genetic backgrounds.

RESOURCE AVAILABILITY

Lead contact

Further information and requests for resources and reagents should be directed to and will be fulfilled by the lead contact, Nicholas J. Maragakis (nmaragak@jhmi.edu), Professor of Neurology, Johns Hopkins University.

Materials availability

This study did not generate new, unique reagents, cell lines, or organisms.

Data and code availability

- This study did not generate new datasets.
- This study did not report original code.
- Any additional information required to reanalyze the data reported in this paper is available from the lead contact upon request.

ACKNOWLEDGMENTS

This manuscript was supported by the National Institute of Health (5R01NS117604, R25NS065729 [to A.T], and K08NS102526), a Maryland Stem Cell Research funding grant (2023-MSCRFD-6125 to N.J.M.), and a Doris Duke Foundation Clinical Scientist Award (to C.W.H.)

AUTHOR CONTRIBUTIONS

A.C. designed the study, performed the majority of the experiments, and wrote the manuscript. A.T. contributed to experimental design, conducted immunohistochemistry, and assisted in manuscript writing and review. K.R. and A.M. contributed to cell culture and iPSC differentiation. K.M. contributed to the design of the study and experiments, conducted immunohistochemistry, and helped perform initial experiments that informed the study. E.L., E.M., and R.S. contributed to data analysis. Y.Z., C.O., and T.-H.W. designed and fabricated the photomasks and assisted in the generation of the microfluidic devices. A.V. contributed materials and assisted in the fabrication and bonding of the microfluidic devices onto MEA plates. C.W.H. contributed to the design and execution of electrophysiological experiments and assisted in manuscript review and writing. N.J.M. supervised the project, contributed to study design, and edited the manuscript.

DECLARATION OF INTERESTS

The authors declare no competing interests.

STAR★METHODS

Detailed methods are provided in the online version of this paper and include the following:

- KEY RESOURCES TABLE
- EXPERIMENTAL MODEL AND STUDY PARTICIPANT DETAILS
  - Human iPSCs and region-specific astrocyte sources
- METHOD DETAILS
  - Differentiating cortical NPCs from hiPSCs
  - Differentiating cortical neurons and astrocytes from cortical NPCs
  - Differentiating spinal NPCs from hiPSCs
  - Differentiating spinal neurons and astrocytes from spinal NPCs
  - Generation of region-specific co-cultures
  - Microfluidic device fabrication
  - Cell migration assessments
  - Media exchange assessment
  - Immunocytochemistry
  - *Trans*-synaptic tracing with rabies virus
  - MEA electrophysiological recordings
- QUANTIFICATION AND STATISTICAL ANALYSIS
  - Quantitative analysis of neurite outgrowth
  - Statistical analysis

SUPPLEMENTAL INFORMATION

Supplemental information can be found online at https://doi.org/10.1016/j.crmeth.2026.101457.

Received: June 27, 2025
Revised: January 29, 2026
Accepted: April 21, 2026
Published: May 19, 2026

REFERENCES

1. Menon, P., and Vucic, S. (2021). The Upper Motor Neuron—Improved Knowledge from ALS and Related Clinical Disorders. Brain Sci. *11*, 958.
2. Gosset, P., Camu, W., Raoul, C., and Mezghrani, A. (2022). Prionoids in amyotrophic lateral sclerosis. Brain Commun. *4*, fcac145.
3. McAlary, L., Chew, Y.L., Lum, J.S., Geraghty, N.J., Yerbury, J.J., and Cashman, N.R. (2020). Amyotrophic lateral sclerosis: proteins, proteostasis, prions, and promises. Front. Cell. Neurosci. *14*, 581907.
4. Lemon, R.N. (2008). Descending pathways in motor control. Annu. Rev. Neurosci. *31*, 195–218. https://doi.org/10.1146/annurev.neuro.31.060407.125547.
5. Roze, E., Dubacq, C., and Welniarz, Q. (2025). Corticospinal Tract Development, Evolution, and Skilled Movements. Mov. Disord. *40*, 1221–1232. https://doi.org/10.1002/mds.30199.

CellPress
OPEN ACCESS

6. Wieters, F., Weiss Lucas, C., Gruhn, M., Büschges, A., Fink, G.R., and Aswendt, M. (2021). Introduction to spasticity and related mouse models. Exp. Neurol. *335*, 113491. https://doi.org/10.1016/j.expneurol.2020.113491.

7. Bonifacino, T., Zerbo, R.A., Balbi, M., Torazza, C., Frumento, G., Fedele, E., Bonanno, G., and Milanese, M. (2021). Nearly 30 Years of Animal Models to Study Amyotrophic Lateral Sclerosis: A Historical Overview and Future Perspectives. Int. J. Mol. Sci. *22*, 12236. https://doi.org/10.3390/ijms222212236.

8. Reale, L.A., Dyer, M.S., Perry, S.E., Young, K.M., Dickson, T.C., Woodhouse, A., and Blizzard, C.A. (2023). Pathologically mislocalised TDP-43 in upper motor neurons causes a die-forward spread of ALS-like pathogenic changes throughout the mouse corticomotor system. Prog. Neurobiol. *226*, 102449. https://doi.org/10.1016/j.pneurobio.2023.102449.

9. Odierna, G.L., Vucic, S., Dyer, M., Dickson, T., Woodhouse, A., and Blizzard, C. (2024). How do we get from hyperexcitability to excitotoxicity in amyotrophic lateral sclerosis? Brain *147*, 1610–1621. https://doi.org/10.1093/brain/awae039.

10. Ozdinler, P.H., Benn, S., Yamamoto, T.H., Güzel, M., Brown, R.H., Jr., and Macklis, J.D. (2011). Corticospinal motor neurons and related subcerebral projection neurons undergo early and specific neurodegeneration in hSOD1G(9)(3)A transgenic ALS mice. J. Neurosci. *31*, 4166–4177. https://doi.org/10.1523/JNEUROSCI.4184-10.2011.

11. Deng, H.X., Zhai, H., Fu, R., Shi, Y., Gorrie, G.H., Yang, Y., Liu, E., Dal Canto, M.C., Mugnaini, E., and Siddique, T. (2007). Distal axonopathy in an alsin-deficient mouse model. Hum. Mol. Genet. *16*, 2911–2920. https://doi.org/10.1093/hmg/ddm251.

12. Fil, D., DeLoach, A., Yadav, S., Alkam, D., MacNicol, M., Singh, A., Compadre, C.M., Goellner, J.J., O'Brien, C.A., Fahmi, T., et al. (2017). Mutant Profilin1 transgenic mice recapitulate cardinal features of motor neuron disease. Hum. Mol. Genet. *26*, 686–701. https://doi.org/10.1093/hmg/ddw429.

13. Kim, J., Hughes, E.G., Shetty, A.S., Arlotta, P., Goff, L.A., Bergles, D.E., and Brown, S.P. (2017). Changes in the Excitability of Neocortical Neurons in a Mouse Model of Amyotrophic Lateral Sclerosis Are Not Specific to Corticospinal Neurons and Are Modulated by Advancing Disease. J. Neurosci. *37*, 9037–9053. https://doi.org/10.1523/JNEUROSCI.0811-17.2017.

14. de Noordhout, A.M., Rapisarda, G., Bogacz, D., Gérard, P., De Pasqua, V., Pennisi, G., and Delwaide, P.J. (1999). Corticomotoneuronal synaptic connections in normal man: an electrophysiological study. Brain *122*, 1327–1340. https://doi.org/10.1093/brain/122.7.1327.

15. Lemon, R.N. (2021). The Cortical "Upper Motoneuron" in Health and Disease. Brain Sci. *11*, 619. https://doi.org/10.3390/brainsci11050619.

16. Takasawa, E., Abe, M., Chikuda, H., and Hanakawa, T. (2022). A computational model based on corticospinal functional MRI revealed asymmetrically organized motor corticospinal networks in humans. Commun. Biol. *5*, 664. https://doi.org/10.1038/s42003-022-03615-2.

17. Benatar, M. (2007). Lost in translation: treatment trials in the SOD1 mouse and in human ALS. Neurobiol. Dis. *26*, 1–13.

18. Maragakis, N.J., de Carvalho, M., and Weiss, M.D. (2023). Therapeutic targeting of ALS pathways: Refocusing an incomplete picture. Ann. Clin. Transl. Neurol. *10*, 1948–1971. https://doi.org/10.1002/acn3.51887.

19. Taga, A., Dastgheyb, R., Habela, C., Joseph, J., Richard, J.-P., Gross, S.K., Lauria, G., Lee, G., Haughey, N., and Maragakis, N.J. (2019). Role of human-induced pluripotent stem cell-derived spinal cord astrocytes in the functional maturation of motor neurons in a multielectrode array system. Stem Cells Transl. Med. *8*, 1272–1285.

20. Ristanović, D., Milosević, N.T., and Stulić, V. (2006). Application of modified Sholl analysis to neuronal dendritic arborization of the cat spinal cord. J. Neurosci. Methods *158*, 212–218. https://doi.org/10.1016/j.jneumeth.2006.05.030.

21. Damiani, D., Baggiani, M., Della Vecchia, S., Naef, V., and Santorelli, F.M. (2024). Pluripotent Stem Cells as a Preclinical Cellular Model for Studying Hereditary Spastic Paraplegias. Int. J. Mol. Sci. *25*, 2615. https://doi.org/10.3390/ijms25052615.

22. Buchner, F., Dokuzluoglu, Z., Grass, T., and Rodriguez-Muela, N. (2023). Spinal cord organoids to study motor neuron development and disease. Life *13*, 1254.

23. Giacomelli, E., Vahsen, B.F., Calder, E.L., Xu, Y., Scaber, J., Gray, E., Dafinca, R., Talbot, K., and Studer, L. (2022). Human stem cell models of neurodegeneration: From basic science of amyotrophic lateral sclerosis to clinical translation. Cell Stem Cell *29*, 11–35. https://doi.org/10.1016/j.stem.2021.12.008.

24. Acharya, P., Choi, N.Y., Shrestha, S., Jeong, S., and Lee, M.Y. (2024). Brain organoids: A revolutionary tool for modeling neurological disorders and development of therapeutics. Biotechnol. Bioeng. *121*, 489–506. https://doi.org/10.1002/bit.28606.

25. Centeno, E.G.Z., Cimarosti, H., and Bithell, A. (2018). 2D versus 3D human induced pluripotent stem cell-derived cultures for neurodegenerative disease modelling. Mol. Neurodegener. *13*, 27.

26. Kim, H., Kim, G.S., Hyun, S.-H., and Kim, E. (2023). Advancements in 2D and 3D In Vitro Models for Studying Neuromuscular Diseases. Int. J. Mol. Sci. *24*, 17006.

27. Nebol, A., and Gouti, M. (2024). A new era in neuromuscular junction research: current advances in self-organized and assembled in vitro models. Curr. Opin. Genet. Dev. *87*, 102229.

28. Salzinger, A., Ramesh, V., Das Sharma, S., Chandran, S., and Thangaraj Selvaraj, B. (2024). Neuronal Circuit Dysfunction in Amyotrophic Lateral Sclerosis. Cells *13*, 792.

29. Liu, R., Meng, X., Yu, X., Wang, G., Dong, Z., Zhou, Z., Qi, M., Yu, X., Ji, T., and Wang, F. (2022). From 2D to 3D co-culture systems: a review of co-culture models to study the neural cells interaction. Int. J. Mol. Sci. *23*, 13116.

30. Iyer, N.R., and Ashton, R.S. (2022). Bioengineering the human spinal cord. Front. Cell Dev. Biol. *10*, 942742. https://doi.org/10.3389/fcell.2022.942742.

31. Gois, A.M., Mendonça, D.M.F., Freire, M.A.M., and Santos, J.R. (2020). In vitro and in vivo models of amyotrophic lateral sclerosis: an updated overview. Brain Res. Bull. *159*, 32–43.

32. Li, X., Tao, Y., Bradley, R., Du, Z., Tao, Y., Kong, L., Dong, Y., Jones, J., Yan, Y., Harder, C.R.K., et al. (2018). Fast Generation of Functional Subtype Astrocytes from Human Pluripotent Stem Cells. Stem Cell Rep. *11*, 998–1008. https://doi.org/10.1016/j.stemcr.2018.08.019.

33. Bradley, R.A., Shireman, J., McFalls, C., Choi, J., Canfield, S.G., Dong, Y., Liu, K., Lisota, B., Jones, J.R., Petersen, A., et al. (2019). Regionally specified human pluripotent stem cell-derived astrocytes exhibit different molecular signatures and functional properties. Development *146*, dev170910. https://doi.org/10.1242/dev.170910.

34. Arlotta, P., Molyneaux, B.J., Chen, J., Inoue, J., Kominami, R., and Macklis, J.D. (2005). Neuronal subtype-specific genes that control corticospinal motor neuron development in vivo. Neuron *45*, 207–221. https://doi.org/10.1016/j.neuron.2004.12.036.

35. Boulting, G.L., Kiskinis, E., Croft, G.F., Amoroso, M.W., Oakley, D.H., Wainger, B.J., Williams, D.J., Kahler, D.J., Yamaki, M., Davidow, L., et al. (2011). A functionally characterized test set of human induced pluripotent stem cells. Nat. Biotechnol. *29*, 279–286. https://doi.org/10.1038/nbt.1783.

36. Wen, Z., Nguyen, H.N., Guo, Z., Lalli, M.A., Wang, X., Su, Y., Kim, N.S., Yoon, K.J., Shin, J., Zhang, C., et al. (2014). Synaptic dysregulation in a human iPS cell model of mental disorders. Nature *515*, 414–418. https://doi.org/10.1038/nature13716.

37. Roybon, L., Lamas, N.J., Garcia, A.D., Yang, E.J., Sattler, R., Lewis, V.J., Kim, Y.A., Kachel, C.A., Rothstein, J.D., Przedborski, S., et al. (2013). Human stem cell-derived spinal cord astrocytes with defined mature or

reactive phenotypes. Cell Rep. *4*, 1035–1048. https://doi.org/10.1016/j.celrep.2013.06.021.

38. Taga, A., Habela, C.W., Johns, A., Liu, S., O'Brien, M., and Maragakis, N.J. (2021). Establishment of an Electrophysiological Platform for Modeling ALS with Regionally-Specific Human Pluripotent Stem Cell-Derived Astrocytes and Neurons. J. Vis. Exp. https://doi.org/10.3791/62726.
39. Salimpour, Y., Anderson, W.S., Dastgheyb, R., Liu, S., Ming, G.L., Song, H., Maragakis, N.J., and Habela, C.W. (2024). Phase-amplitude coupling detection and analysis of human 2-dimensional neural cultures in multiwell microelectrode array in vitro. J. Neurosci. Methods *407*, 110127. https://doi.org/10.1016/j.jneumeth.2024.110127.
40. Habela, C.W., Liu, S., Taga, A., Oddoye, S., Dastgheyb, R., Haughey, N., Bergles, D.E., Song, H., Ming, G.L., and Maragakis, N.J. (2025). Altered development and network connectivity in a human neuronal model of 15q11.2 deletion-related neurodevelopmental disorders. Transl. Psychiatry *15*, 329. https://doi.org/10.1038/s41398-025-03453-w.
41. Callaway, E.M., and Luo, L. (2015). Monosynaptic Circuit Tracing with Glycoprotein-Deleted Rabies Viruses. J. Neurosci. *35*, 8979–8985. https://doi.org/10.1523/JNEUROSCI.0409-15.2015.
42. Andersen, J., Revah, O., Miura, Y., Thom, N., Amin, N.D., Kelley, K.W., Singh, M., Chen, X., Thete, M.V., Walczak, E.M., et al. (2020). Generation of Functional Human 3D Cortico-Motor Assembloids. Cell *183*, 1913–1929.e26. https://doi.org/10.1016/j.cell.2020.11.017.
43. Gois, A.M., Mendonça, D.M.F., Freire, M.A.M., and Santos, J.R. (2020). In Vitro and in Vivo Models of Amyotrophic Lateral Sclerosis: An Updated Overview. Brain Res. Bull. *159*, 32–43. https://doi.org/10.1016/j.brainresbull.2020.03.012.
44. Tan, R.H., Ke, Y.D., Ittner, L.M., and Halliday, G.M. (2017). ALS/FTLD: experimental models and reality. Acta Neuropathol. *133*, 177–196. https://doi.org/10.1007/s00401-016-1666-6.
45. Lemon, R. (2024). The Corticospinal System and Amyotrophic Lateral Sclerosis: IFCN handbook chapter. Clin. Neurophysiol. *160*, 56–67. https://doi.org/10.1016/j.clinph.2024.02.001.
46. Welniarz, Q., Dusart, I., and Roze, E. (2017). The corticospinal tract: Evolution, development, and human disorders. Dev. Neurobiol. *77*, 810–829. https://doi.org/10.1002/dneu.22455.
47. Okano, H., and Morimoto, S. (2022). iPSC-based disease modeling and drug discovery in cardinal neurodegenerative disorders. Cell Stem Cell *29*, 189–208. https://doi.org/10.1016/j.stem.2022.01.007.
48. Beghini, D.G., Kasai-Brunswick, T.H., and Henriques-Pons, A. (2024). Induced Pluripotent Stem Cells in Drug Discovery and Neurodegenerative Disease Modelling. Int. J. Mol. Sci. *25*, 2392. https://doi.org/10.3390/ijms25042392.
49. Clayton, B.L.L., Barbar, L., Sapar, M., Kalpana, K., Rao, C., Migliori, B., Rusielewicz, T., , et al.; NYSCF Global Stem Cell Array® Team; Paull, D., Brenner, K. (2024). Patient iPSC models reveal glia-intrinsic phenotypes in multiple sclerosis. Cell Stem Cell *31*, 1701–1713.e8. https://doi.org/10.1016/j.stem.2024.08.002.
50. Mutukula, N., Man, Z., Takahashi, Y., Iniesta Martinez, F., Morales, M., Carreon-Guarnizo, E., Hernandez Clares, R., Garcia-Bernal, D., Martinez Martinez, L., Lajara, J., et al. (2021). Generation of RRMS and PPMS specific iPSCs as a platform for modeling Multiple Sclerosis. Stem Cell Res. *53*, 102319. https://doi.org/10.1016/j.scr.2021.102319.
51. Plastini, M.J., Desu, H.L., Ascona, M.C., Lang, A.L., Saporta, M.A., and Brambilla, R. (2022). Transcriptional abnormalities in induced pluripotent stem cell-derived oligodendrocytes of individuals with primary progressive multiple sclerosis. Front. Cell. Neurosci. *16*, 972144. https://doi.org/10.3389/fncel.2022.972144.
52. Endo, F., Kasai, A., Soto, J.S., Yu, X., Qu, Z., Hashimoto, H., Gradinaru, V., Kawaguchi, R., and Khakh, B.S. (2022). Molecular basis of astrocyte diversity and morphology across the CNS in health and disease. Science *378*, eadc9020. https://doi.org/10.1126/science.adc9020.
53. Spurgat, M.S., and Tang, S.J. (2022). Single-Cell RNA-Sequencing: Astrocyte and Microglial Heterogeneity in Health and Disease. Cells *11*, 2021. https://doi.org/10.3390/cells11132021.
54. Torres-Ceja, B., and Olsen, M.L. (2022). A closer look at astrocyte morphology: Development, heterogeneity, and plasticity at astrocyte leaflets. Curr. Opin. Neurobiol. *74*, 102550. https://doi.org/10.1016/j.conb.2022.102550.
55. Buosi, A.S., Matias, I., Araujo, A.P.B., Batista, C., and Gomes, F.C.A. (2018). Heterogeneity in Synaptogenic Profile of Astrocytes from Different Brain Regions. Mol. Neurobiol. *55*, 751–762. https://doi.org/10.1007/s12035-016-0343-z.
56. Kempf, J., Knelles, K., Hersbach, B.A., Petrik, D., Riedemann, T., Bednarova, V., Janjic, A., Simon-Ebert, T., Enard, W., Smialowski, P., et al. (2021). Heterogeneity of neurons reprogrammed from spinal cord astrocytes by the proneural factors Ascl1 and Neurogenin2. Cell Rep. *36*, 109571. https://doi.org/10.1016/j.celrep.2021.109571.
57. Fossati, G., Matteoli, M., and Menna, E. (2020). Astrocytic Factors Controlling Synaptogenesis: A Team Play. Cells *9*, 2173. https://doi.org/10.3390/cells9102173.
58. Hasel, P., Dando, O., Jiwaji, Z., Baxter, P., Todd, A.C., Heron, S., Márkus, N.M., McQueen, J., Hampton, D.W., Torvell, M., et al. (2018). Author Correction: Neurons and neuronal activity control gene expression in astrocytes to regulate their development and metabolism. Nat. Commun. *9*, 16176. https://doi.org/10.1038/ncomms16176.
59. Van Horn, M.R., Benfey, N.J., Shikany, C., Severs, L.J., and Deemyad, T. (2021). Neuron-astrocyte networking: astrocytes orchestrate and respond to changes in neuronal network activity across brain states and behaviors. J. Neurophysiol. *126*, 627–636. https://doi.org/10.1152/jn.00062.2021.
60. Pathak, D., and Sriram, K. (2023). Neuron-astrocyte omnidirectional signaling in neurological health and disease. Front. Mol. Neurosci. *16*, 1169320. https://doi.org/10.3389/fnmol.2023.1169320.
61. Buchner, F., Dokuzluoglu, Z., Grass, T., and Rodriguez-Muela, N. (2023). Spinal Cord Organoids to Study Motor Neuron Development and Disease. Life *13*, 1254. https://doi.org/10.3390/life13061254.
62. Kim, J.I., Imaizumi, K., Thete, M.V., Hudacova, Z., Jurjuţ, O., Amin, N.D., Scherrer, G., and Paşca, S.P. (2025). Human assembloid model of the ascending neural sensory pathwayNature.*642*, 143–153. https://doi.org/10.1038/s41586-025-08808-3
63. Amartumur, S., Nguyen, H., Huynh, T., Kim, T.S., Woo, R.S., Oh, E., Kim, K.K., Lee, L.P., and Heo, C. (2024). Neuropathogenesis-on-chips for neurodegenerative diseases. Nat. Commun. *15*, 2219. https://doi.org/10.1038/s41467-024-46554-8.
64. Leng, Y., Li, X., Zheng, F., Liu, H., Wang, C., Wang, X., Liao, Y., Liu, J., Meng, K., Yu, J., et al. (2023). Advances in In Vitro Models of Neuromuscular Junction: Focusing on Organ-on-a-Chip, Organoids, and Biohybrid Robotics. Adv. Mater. *35*, e2211059. https://doi.org/10.1002/adma.202211059.
65. Dauth, S., Maoz, B.M., Sheehy, S.P., Hemphill, M.A., Murty, T., Macedonia, M.K., Greer, A.M., Budnik, B., and Parker, K.K. (2017). Neurons derived from different brain regions are inherently different in vitro: a novel multiregional brain-on-a-chip. J. Neurophysiol. *117*, 1320–1341. https://doi.org/10.1152/jn.00575.2016.
66. Dauth, S., Grevesse, T., Pantazopoulos, H., Campbell, P.H., Maoz, B.M., Berretta, S., and Parker, K.K. (2016). Extracellular matrix protein expression is brain region dependent. J. Comp. Neurol. *524*, 1309–1336. https://doi.org/10.1002/cne.23965.
67. Sherwood, C.C., Stimpson, C.D., Raghanti, M.A., Wildman, D.E., Uddin, M., Grossman, L.I., Goodman, M., Redmond, J.C., Bonar, C.J., Erwin, J.M., and Hof, P.R. (2006). Evolution of increased glia-neuron ratios in the human frontal cortex. Proc. Natl. Acad. Sci. USA *103*, 13606–13611. https://doi.org/10.1073/pnas.0605843103.
68. Ruiz-Sauri, A., Orduña-Valls, J.M., Blasco-Serra, A., Tornero-Tornero, C., Cedeño, D.L., Bejarano-Quisoboni, D., Valverde-Navarro, A.A., Benyamin,

R., and Vallejo, R. (2019). Glia to neuron ratio in the posterior aspect of the human spinal cord at thoracic segments relevant to spinal cord stimulation. J. Anat. *235*, 997–1006. https://doi.org/10.1111/joa.13061.

69. Tong, Z., Kwak, E., Aguiar, A., Peng, B., Pouton, C.W., Voelcker, N.H., and Haynes, J.M. (2021). Compartmentalized microfluidic chambers enable long-term maintenance and communication between human pluripotent stem cell-derived forebrain and midbrain neurons. Lab Chip *21*, 4016–4030. https://doi.org/10.1039/d1lc00505g.

70. Quintanilla, C.A., Fitzgerald, Z., Kashow, O., Radojicic, M.S., Ulupinar, E., Bitlis, D., Genc, B., Andjus, P., van Drongelen, W., and Ozdinler, P.H. (2025). High-density multielectrode arrays bring cellular resolution to neuronal activity and network analyses of corticospinal motor neurons. Sci. Rep. *15*, 732. https://doi.org/10.1038/s41598-024-83883-6.

71. Miura, Y., Li, M.Y., Birey, F., Ikeda, K., Revah, O., Thete, M.V., Park, J.Y., Puno, A., Lee, S.H., Porteus, M.H., and Pașca, S.P. (2020). Generation of human striatal organoids and cortico-striatal assembloids from human pluripotent stem cells. Nat. Biotechnol. *38*, 1421–1430. https://doi.org/10.1038/s41587-020-00763-w.

72. Johns, A.E., Taga, A., Charalampopoulou, A., Gross, S.K., Rust, K., McCray, B.A., Sullivan, J.M., and Maragakis, N.J. (2024). Exploring P2X7 receptor antagonism as a therapeutic target for neuroprotection in an hiPSC motor neuron model. Stem Cells Transl. Med. *13*, 1198–1212. https://doi.org/10.1093/stcltm/szae074.

73. Li, W., Sun, W., Zhang, Y., Wei, W., Ambasudhan, R., Xia, P., Talantova, M., Lin, T., Kim, J., Wang, X., et al. (2011). Rapid induction and long-term self-renewal of primitive neural precursors from human embryonic stem cells by small molecule inhibitors. Proc. Natl. Acad. Sci. USA *108*, 8299–8304. https://doi.org/10.1073/pnas.1014041108.

74. Page, S.C., Sripathy, S.R., Farinelli, F., Ye, Z., Wang, Y., Hiler, D.J., Pattie, E.A., Nguyen, C.V., Tippani, M., Moses, R.L., et al. (2022). Electrophysiological measures from human iPSC-derived neurons are associated with schizophrenia clinical status and predict individual cognitive performance. Proc. Natl. Acad. Sci. USA *119*, e2109395119. https://doi.org/10.1073/pnas.2109395119.

75. O'Keefe, C.M., Pisanic, T.R., 2nd, Zec, H., Overman, M.J., Herman, J.G., and Wang, T.H. (2018). Facile profiling of molecular heterogeneity by microfluidic digital melt. Sci. Adv. *4*, eaat6459. https://doi.org/10.1126/sciadv.aat6459.

76. Zhao, Y., O'Keefe, C.M., Hu, J., Allan, C.M., Cui, W., Lei, H., Chiu, A., Hsieh, K., Joyce, S.C., Herman, J.G., et al. (2024). Multiplex digital profiling of DNA methylation heterogeneity for sensitive and cost-effective cancer detection in low-volume liquid biopsies. Sci. Adv. *10*, eadp1704. https://doi.org/10.1126/sciadv.adp1704.

77. Longair, M.H., Baker, D.A., and Armstrong, J.D. (2011). Simple Neurite Tracer: open source software for reconstruction, visualization and analysis of neuronal processes. Bioinformatics *27*, 2453–2454. https://doi.org/10.1093/bioinformatics/btr390.

STAR★METHODS

KEY RESOURCES TABLE

REAGENT or RESOURCE	SOURCE	IDENTIFIER
Antibodies		
Rabbit Anti-βIII tubulin	Millipore Sigma	T3952
Mouse Anti- βIII tubulin	Abcam	AB78078
Chicken Anti- βIII tubulin	Millipore Sigma	AB9354
Mouse ISL 1/2	Developmental Studies Hybridoma Bank	39.4D5, concentrate
Goat ChaT	Millipore Sigma	AB144P
Chicken Anti-GFP	Aves Labs	GFP-1020
Mouse VGLUT1	Synaptic Systems	135311
Rabbit SATB2	Abcam	AB92446
Rat CTIP2	Abcam	AB18465
Mouse GAD67	Millipore Sigma	MAB5406
Rabbit CAMKIIa	Thermo Fisher Scientific	PA5-14315
Mouse S100B	Millipore Sigma	ABN59
Chicken GFAP	Millipore sigma	AB5541
Rat HOXB4	Developmental Studies Hybridoma Bank	I12, concentrate
Mouse OTX2	Invitrogen	MA5-15854
Hoechst 33342	Millipore Sigma	34580
Alexafluor^{TM} Goat anti-chicken 488	ThermoFisher Scientific	A11039
Alexafluor^{TM} Goat anti-chicken 647	ThermoFisher Scientific	A21449
Alexafluor^{TM} Goat anti-rabbit 488	ThermoFisher Scientific	A11034
Alexafluor^{TM} Goat anti-rabbit 555	ThermoFisher Scientific	A21428
Alexafluor^{TM} Goat anti-rabbit 647	ThermoFisher Scientific	A21245
Alexafluor^{TM} Goat anti-rat 555	ThermoFisher Scientific	A21434
Alexafluor^{TM} Goat anti-mouse 488	ThermoFisher Scientific	A11029
Alexafluor^{TM} Goat anti-mouse 555	ThermoFisher Scientific	A21424
Alexafluor^{TM} Goat anti-mouse 647	ThermoFisher Scientific	A21236
Alexafluor^{TM} Donkey anti-mouse 555	ThermoFisher Scientific	A23424
Alexafluor^{TM} Donkey anti-mouse 647	ThermoFisher Scientific	A31571
Alexafluor^{TM} Donkey anti-rabbit 555	ThermoFisher Scientific	A31572
Alexafluor^{TM} Donkey anti-goat 647	ThermoFisher Scientific	A21447
Alexafluor^{TM} 488 conjugated affiniPure donkey anti-chicken	Jackson labs	703-545-155
Rhodamine red-X-conjugated affiniPure donkey anti-rat IgG	Jackson labs	712-295-153
Bacterial and virus strains		
G-deleted Cre-EGFP Rabies Virus	Miura et al.[71]	N/A
AAV-CAG-FLEX-oG-WPRE-SV40-PA	Addgene (Watertown, MA, USA)	Cat#74292
AAV9 CAMKII-DIO-mCherry	Biohippo, Inc, Gaithersburg, Maryland	Cat#BHV17100457
CAMKII-ChR2-YFP AAV9	Addgene (Watertown, MA, USA)	Cat#26969-AAV9
AAV9-Syn-ChrimsonR-tdT	Addgene (Watertown, MA, USA)	Cat#59171-AAV9
Chemicals, peptides, and recombinant proteins		
NBQX (AMPA/kainate receptor antagonist; 2,3-dioxo-6-nitro-7-sulfamoyl-benzo[f]quinoxaline)	Sigma Aldrich, St. Louis, MO, USA	Cat#N171

(Continued on next page)

Continued		
REAGENT or RESOURCE	SOURCE	IDENTIFIER
APV (NMDA receptor antagonist; 2-Amino-5-phosphonovaleric acid)	Sigma Aldrich, St. Louis, MO, USA	Cat#A5282
488-NucSpot	Biotium	Cat#40081
PDMS and cross-linker (SylgardTM 184)	Electron Microscopy Sciences, Pennsylvania	Cat#24236-10
Critical commercial assays		
Maestro Edge system	Axion Biosystems	https://www.axionbiosystems.com/products/mea/maestro-edge
Lumos (Optogenetic stimulation)	Axion Biosystems	https://www.axionbiosystems.com/products/mea/maestro-edge
Experimental models: Cell lines		
human iPSC line, CS9XH7	Cedars Sinai repository	CS9XH7iCTR-nxx
human iPSC cortical Astrocytes, WC-30	BrainXell	Cat#BX-0600-30
human iPSC spinal Astrocytes, WC-30	BrainXell	Cat#BX-0650-30
Software and algorithms		
Neural Metric Tool	Axion Biosystems	https://www.axionbiosystems.com/products/mea/maestro-edge
Axis Navigator	Axion Biosystems	https://www.axionbiosystems.com/products/mea/maestro-edge
Graph Pad Prism software 10.4.1	Boston, MA, USA	https://www.graphpad.com/scientific-software/prism/
Imaris software	Oxford Instruments, Oxon, UK	https://imaris.oxinst.com/
ZEN 3.6 Blue edition software	Carl Zeiss Microscopy GmbH, Jena, Germany	https://www.zeiss.com/microscopy/en/products/software/zen.html
Fiji package of ImageJ	Fiji/ImageJ community (LOCI, UW–Madison)	https://fiji.sc/

EXPERIMENTAL MODEL AND STUDY PARTICIPANT DETAILS

Human iPSCs and region-specific astrocyte sources

hiPSC-derived Cortical neurons and spinal motor neurons were differentiated from a single healthy control (53yo male) hiPSC line (CS9XH7; Cedars-Sinai, catalog CS9XH7iCTR-Nxx). Using one donor line for both neuronal subtypes minimized inter-line variability. Region-specific hiPSC-derived astrocytes were incorporated from a healthy control line (WC-30) using commercially available, previously characterized products: cortical astrocytes (forebrain-patterned; BrainXell BX-0600) and spinal astrocytes (spinal-patterned; BrainXell BX-0650).

METHOD DETAILS

Differentiating cortical NPCs from hiPSCs

Human iPSC derived cortical NPCs were generated from a control line (CS9XH7) using a human forebrain cortical differentiation protocol adapted from Wen et al. 2014,[36] and as previously published by our group.[40,72] Briefly, iPSC colonies were detached from the Matrigel (Corning) with 1 mg/mL collagenase for 1h and suspended in embryoid body (EB) medium, consisting of DMEM/F12 (−) L-Glutamine, 20% knockout serum replacement (KSR), penicillin-streptomycin (Pen/Strep), GlutaMax, non-essential amino acids (NEAA), supplemented with [55μM] β-Mercaptoethanol (Gibco), [2μM] dorsomorphin, and [2μM] A-83, in low-attachment plates (Corning) plates for 4 days with daily medium exchanges. After 4 days *in vitro* (DIV), EB medium was replaced by NPC induction medium (“NPC medium”) consisting of DMEM/F12, N2 supplement, NEAA, 2μg/mL heparin and [2μM] cyclopamine. The floating EBs were then transferred to Matrigel-coated 6-well plates on day 7 and allowed to form neural rosettes. The attached rosettes were maintained for 15days in NPC medium, changed three times per week. After 22DIV, neural rosettes were selected and lifted mechanically after incubation with neural rosette selection reagent (StemDiff, STEMCELL technologies) for 1 h and transferred to low attachment plates (Corning) in NPC medium enriched with B27 supplement (Gibco). Resuspended neural progenitor spheres were dissociated with accutase (Gibco) at 37°C for 10 min and frozen in “Cortical Neuronal Medium” consisting of Neurobasal base (Gibco) with B27, NEAA, GlutaMax, 10 ng/mL BDNF, 10 ng/mL GDNF and supplemented with 10% DMSO for cryopreservation.

Differentiating cortical neurons and astrocytes from cortical NPCs

After 23 DIV, cortical NPCs were thawed and plated onto plates that had been first treated with Poly-L-ornithine (PLO) diluted in water for 1 h at 37°C and then coated with 1:100 mouse laminin (1μg/mL, Gibco). Frozen NPCs were flash thawed in a 37°C water bath, centrifuged and resuspended in 10 mL of cortical neuronal media with 20μM of ROCK-I (1:500 DMSO) and added to the laminin coated plates. For cortical neuron maturation, during the first week after plating, neuronal cultures were treated for 48 h with 0.2μM cytosine arabinoside (ARA-C) in neuronal media to suppress astrocyte/glial proliferation. Cells were maintained with full media changes three times a week from DIV23 to DIV44. The media consisted of cortical neuronal medium supplemented with [125nM] Compound E (Santa Cruz Biotechnology) to further enhance motor neuronal maturation.[73]

Human iPSC-derived cortical astrocytes derived from a separate control hiPSC line (WC-30) were purchased from BrainXell and differentiated per manufacturer's recommendation for a week. Then, cells were cultured with Astrocyte Differentiation medium with 1% fetal bovine serum (FBS) consisting of DMEM/F12, GlutaMax, NEAA, 1%v/v FBS, Pen/Strep, B27 supplement and 2μg/ml Heparin. Cells were expanded into Matrigel-coated plates and passaged upon reaching confluency until DIV15-25 when they are used for seeding.

Differentiating spinal NPCs from hiPSCs

Induced pluripotent stem cells from the same control line (CS9XH7) were differentiated into spinal NPCs following modifications of a 25-day protocol described previously[35] and previously used by our group,[19] that relies on extrinsic morphogens to pattern NPCs along the rostro-caudal and dorsoventral axis. Briefly, key steps include neuralization via dual SMAD ("Suppressor of Mothers against Decapentaplegic") signaling inhibition using LDN193189 (Stemgent) and SB431542 (Millipore Sigma), followed by caudalization and ventralization through retinoic acid (RA) and purmorphamine (PMN) (Millipore Sigma).

Differentiating spinal neurons and astrocytes from spinal NPCs

25DIV spinal NPCs were thawed and plated onto PLO and Laminin-coated plates as described above for cortical NPCs. For motor neuron differentiation, spinal NPCs were cultured with "spinal neuronal differentiation medium", comprised of Neurobasal (Gibco), enriched with N2 and B27 supplements (Gibco), GlutMax, NEAA, pen/strep and supplemented with growth factors (RA, PMN, ascorbic acid, recombinant human-brain-derived neurotrophic factor, glial cell line-derived neurotrophic factor, insulin-like growth factor 1, ciliary neurotrophic factor). Similar to the cortical differentiation protocol, Compound E (Santa Cruz Biotechnology) was added to enhance neuronal differentiation into motor neurons. To prevent astrocyte over-proliferation, neuronal cultures were treated once between DIV32-35 with 0.02μM Ara-C for 48h. The medium was then changed every other day. At 60 DIV, this protocol has been shown to generate a population of spinal cord neurons, with a majority of neurons expressing spinal motor neuron markers, including ChAT and ISL1/2.[19]

Human iPSC-derived spinal astrocytes (control line WC-30) were purchased from BrainXell and differentiated per manufacturer's recommendation for a week. Cells were then cultured with astrocyte differentiating medium with 1%v/v FBS as described above for cortical astrocytes.

Generation of region-specific co-cultures

The surfaces of the chips were coated with Geltrex overnight. Cortical neurons were first seeded in the cortical compartment, we chemoattracted the axons using media enriched with BDNF (50ng/ml) and GDNF (50ng/ml) in the spinal compartment only, for 4–6 days. Media exchanges were performed with a slightly smaller volume in the spinal compartment than in the cortical compartment, generating a capillary-driven gradient that limits spinal-to-cortical media exchange and promotes directional axonal outgrowth. After ensuring that the cortical axons had occupied the microchannels and reached the spinal compartment, the device was seeded with other cell types to complete the corticospinal-tract-on-a-chip (CA in the cortical compartment and SpMN, SpA in the spinal compartment). Cortical astrocytes were plated on top of cortical neurons. Spinal neurons and spinal astrocytes were mixed and plated simultaneously in the spinal compartment. Cell densities in the chip were $6 \times 10^4/cm^2$ and $8 \times 10^4/cm^2$ (ratio neurons: astrocytes, 2:1), at DIV47 and DIV15-20 for neurons and BrainXell astrocytes, respectively. These densities generated a confluent layer of astrocytes with interspaced neurons, as described previously.[19] In the chip, given the differing media requirements of cortical and spinal motor neuron monocultures, we optimized a composite medium by combining the cortical and the spinal media recipes.[38,39,74] Composite medium for the co-cultures in the devices included Neurobasal: DMEM:F12 at a 50:50 ratio, supplemented with B27 (1:50), N2 (1:100), 10 ng/mL BDNF, 10 ng/mL GDNF, 0.4 μg/mL ascorbic acid and 5%v/v FBS. We performed media exchanges 3 times weekly, by removing 15μL and adding 20μL to each compartment to account for media evaporation.

Microfluidic device fabrication

Microfluidic devices were fabricated using standard photolithography and soft lithography techniques as previously described.[75,76] Photomasks were designed using AutoCAD software and printed by Artnet Pro. Briefly, a silicon wafer was dehydrated by overnight baking at 200°C. The SU-8 3010 photoresist (Kayaku) was spun on the wafer at 3000 rpm to form microchannels layer and SU-8 3050 photoresist was spun subsequently on the wafer at 1000 rpm. The wafer was baked at 95°C for 45 min. After exposure, the wafer was developed and baked at 200°C for an hour. PDMS and cross-linker (Electron Microscopy Sciences, Pennsylvania) were mixed at a 10:1 ratio (w/w), degassed under vacuum, and cast onto the molds to ensure complete coverage of the microchannels. The molds

were cured in an oven at 80°C for 2 h, after which the cured PDMS was carefully peeled off. Square compartments, open at the top to facilitate cell seeding and media addition, were created by punching out the desired regions of the PDMS. The resultant compartmentalized devices consisted of two chambers (surface area of 3.69 mm^2) connected by 100 microchannels with diameters ranging from 8 to 10μm. After sterilization, the devices were bonded by plasma treatment to glass-bottom 12-well plates for imaging studies or to 6-well multielectrode array plates from AxionBiosystem for electrophysiological studies. Prior to cell plating, the surfaces of the box devices were coated with GeltreX (Gibco). Each well accommodates 30 μL of media, providing optimal surface coverage and supporting consistent cell culture conditions.

Cell migration assessments

Cells were seeded into the cortical compartment using the standard protocol described above, and migration into the spinal compartment was evaluated after one week. Given there were no cells in the spinal compartment (opposite compartment from the one where cells were seeded) upon plating, we could identify migrated cells by counting the absolute number of Hoechst+ nuclei in the spinal compartment. Migration data was collected from a total of 6 devices and 3 field images per device were collected. To evaluate cell migration after one month in the corticospinal-tract-on-a-chip, immunostaining was used to detect cortical and spinal region-specific markers in the spinal and cortical compartments, respectively. We stained the cortical compartment for ChAT (a marker of spinal motor neurons) and HOXB4 (a marker of spinal astrocytes), and the spinal compartment for SATB2 (a marker of cortical neurons) and OTX2 (a marker of cortical astrocytes). Immunoreactive cells were quantified as a percentage of the total number of neurons and astrocytes), based on five images per compartment from three separate chips. A minimum of 250 neurons (Hoechst+ TUJ+) and 250 astrocytes (Hoechst+ Tuj-) were counted for percentage calculations.

Media exchange assessment

To assess media exchange and small molecule diffusion across microchannels, we utilized 488-NucSpot 1000X (Biotium), a live nuclear fluorescent dye, as a probe to track small molecule diffusion. Six chips with all cell types seeded were used for this experiment. The live-cell dye was applied to the cortical compartments of three chips, while the remaining three received an equivalent volume of vehicle (DMSO) as control. Live imaging was performed using confocal microscopy at 5x and 10× magnification and diffusion was assessed at various time points. At the 24-h time point, the nuclear dye was also added to the spinal compartments to verify the expected diffusion pattern. Fluorescence intensities were maintained for both compartments at each time point. Images were analyzed using Zeiss Zen software (Carl Zeiss). For representative images, adjacent tiles were acquired with overlapping regions to ensure field continuity and manually aligned into a mosaic using PowerPoint (Microsoft Office 365). Care was taken to preserve spatial continuity and anatomical context between adjacent fields. No image content was altered beyond alignment and cropping.

Immunocytochemistry

Cells were fixed with 4% paraformaldehyde for 10 min and then washed with phosphate-buffered saline (PBS) three times. The cells were then permeabilized with 0.1% Triton X-100 (Millipore Sigma) in PBS for 10 min and washed with PBS three times. A blocking solution with 3% bovine serum albumin (BSA) in PBS was then applied for 1 h. Glass bottom devices were stained with primary antibodies in a blocking solution containing 3% BSA in PBS and 3% species specific serum and incubated overnight at 4°C. The next day, cells were washed with 3% BSA in PBS three times and incubated with appropriate secondary antibodies (Thermo Fisher Scientific, Alexa Fluor Dyes; concentration: 1:1000) and Hoechst (Thermo Fisher Scientific; concentration: 2 μg/mL) in a blocking solution with 3% BSA in PBS and 3% species-specific serum, for 1 h at room temperature. Finally, the coverslips were washed with 3% BSA in PBS three times and mounted with Prolong gold with DAPI (Thermo Fisher Scientific) and stored at 4°C until ready to image. The primary antibodies used for this study are listed in key resources table (KRT).

Images were acquired on a Zeiss fluorescence microscope (Zeiss Airyscan Confocal), using 20x, 40x and 63× oil magnifications and analyzed using Zeiss Zen software. Three to five images were obtained for each compartment of each microfluidic device, and 3 microfluidic devices were utilized for each condition. Cell counts were performed by an individual blinded to the experimental conditions.[19]

Trans-synaptic tracing with rabies virus

hiPSC-derived spinal neurons were transduced with a G-deleted rabies virus expressing Cre recombinase and enhanced green fluorescent protein (EGFP) (Rabies GΔ Cre-EGFP, Salk Institute, La Jolla, California) at DIV50. After a week, the spinal neurons were co-transduced with the helper virus AAV-CAG-FLEX-oG-WPRE-SV40-PA,[71] which expresses the rabies virus glycoprotein (RVG) required for retrograde *trans*-synaptic transfer of Rabies-GFP from the spinal neurons to cortical neurons.

For the cortical compartment, cortical neurons were transduced with an AAV9 vector containing a DIO construct for mCherry expression under the control of the CAMKII promoter (AAV9 CAMKII-DIO-mCherry, Biohippo, Inc, Gaithersburg, Maryland) at DIV44 when seeded at the device to optimize transduction rate. Expression of mCherry was induced by Cre recombinase, which was provided by the Rabies GΔ Cre-EGFP virus, confirming *trans*-synaptic communication. Confocal fluorescence microscopy (oil immersion 40x) was used to detect both EGFP and mCherry signals in the cortical compartment.

MEA electrophysiological recordings

Electrophysiological recordings were conducted using the Maestro Edge system (Axion BioSystems) with bonded microfluidic devices placed on 64-electrode 6-well plates. In each well, 16 to 20 electrodes were located beneath or between the microchannels, where signal artifacts arose due to artificial amplification or due to lack of cell coverage at this part of the surface. These electrodes were excluded from analysis. Plates were pre-coated with Geltrex prior to cell seeding, and electrophysiological parameters were recorded as previously described.[38]

We conducted five-minute weekly recordings of spontaneous activity for a period of 12 weeks. Both basic and synchronous electrophysiological metrics were examined. Voltage measurements were filtered using the neuronal spontaneous activity filter of the software AxisNavigator. Spikes were identified as instantaneous time points of voltages that exceed 5 spikes/sec. In addition to filtering, all traces were visually inspected for quality control. Hypersynchronous (or "network") bursts were defined using the envelope algorithm of the software (Neural Metrics Tool, Axion Biosystems software). To ensure proper electrical connectivity and eliminate recording artifacts caused by the placement of the microfluidic device, the wells were flooded with culture medium to establish a fluidic/electrolyte bridge between the interior and exterior of the device, facilitating communication with the ground electrode. Results were graphed using NeuralMetricTool that generates raster plots of the 300s spontaneous recording. Graphs were generated to depict key electrophysiological metrics over the 12-week experimental period, including weighted mean firing rate (wMFR), number of active electrodes, burst percentage, average burst frequency, network burst frequency, burst duration, synchrony index, and area under the normalized cross-correlation (AUNCC).

For optical stimulation studies, neurotransmitter modulators were tested by applying the AMPA/kainate receptor antagonist NBQX (2,3-dioxo-6-nitro-7-sulfamoyl-benzo[f]quinoxaline, 50uM, Sigma Aldrich, St. Louis, MO, USA) and the NMDA receptor antagonist APV (2-Amino-5-phosphonovaleric acid, 28.4uM). Neurons expressing channelrhodopsin were activated with blue light with wavelength center of 475nm and a maximum intensity of >3.9mW/mm^3. Spinal neurons expressing ChrimsonR (AAV9-Syn-ChrimsonR-tdT, Addgene, Watertown, MA, USA) were activated with orange light and wavelength center of 590nm and the same maximum intensity of >3.9mW/mm^3. Voltage measurements were filtered using the optical stimulation filter in AxisNavigator. Optogenetic stimulation was performed using a modified Axion BioSystems setup, allowing light delivery into the 6-well plates containing the PDMS microfluidic devices. Recordings were 5-min long, with optical stimulation delivered as 5-millisecond (blue or far-red) light pulses at 30% intensity, 0.25 Hz, for a total of 70 stimuli. Events spanning 100 milliseconds of peristimulus time with a bin size of 1 millisecond were analyzed and visualized using Neural Metric Tool (Axion Biosystems).

QUANTIFICATION AND STATISTICAL ANALYSIS

Quantitative analysis of neurite outgrowth

For the morphological analysis of neurons, we used TUJ1 immunostaining and 40× oil magnification images. We first traced the neuronal somatic area manually and tracked individual neurites using the simple neurite tracer software plugin on the Fiji package of ImageJ.[77] We then performed a Sholl analysis on the neurite mask, with ten soma-centered concentric circles of increasing radii (10μm increment).[20] For analysis, 30 neurons were randomly selected per condition, and we calculated the number of intersections/radius and the total sum of intersections for each individual neuron.

Statistical analysis

All data were analyzed using Graph Pad Prism software 10.4.1 (Boston, MA, USA). Data are presented as mean ± SEM. For experiments comparing two conditions over time, we used repeated measures two-way ANOVA when assumptions of normality and equal variances were met. For datasets with small sample sizes or non-normal distributions, we used Wilcoxon signed-rank test on values to assess directional significance. Each experiment included at least three technical replicates. Experimental conditions and technical replicates are specified in the legends. Statistical significance for all tests performed was set at $p < 0.05$.

Cell Reports Methods, Volume *6*

Supplemental information

Development of a human iPSC-derived corticospinal tract-on-a-chip

Andriana Charalampopoulou, Arens Taga, Khalil Rust, Evelyn Luciani, Katherine Marshall, Elliot Montgomery, Anuradha Mansinghka, Richa Singh, Yang Zhao, Christine O'Keefe, Tza-Huei Wang, Arun Venkatesan, Christa Whelan Habela, and Nicholas John Maragakis

A Addition of live cell dye at the cortical compartment only

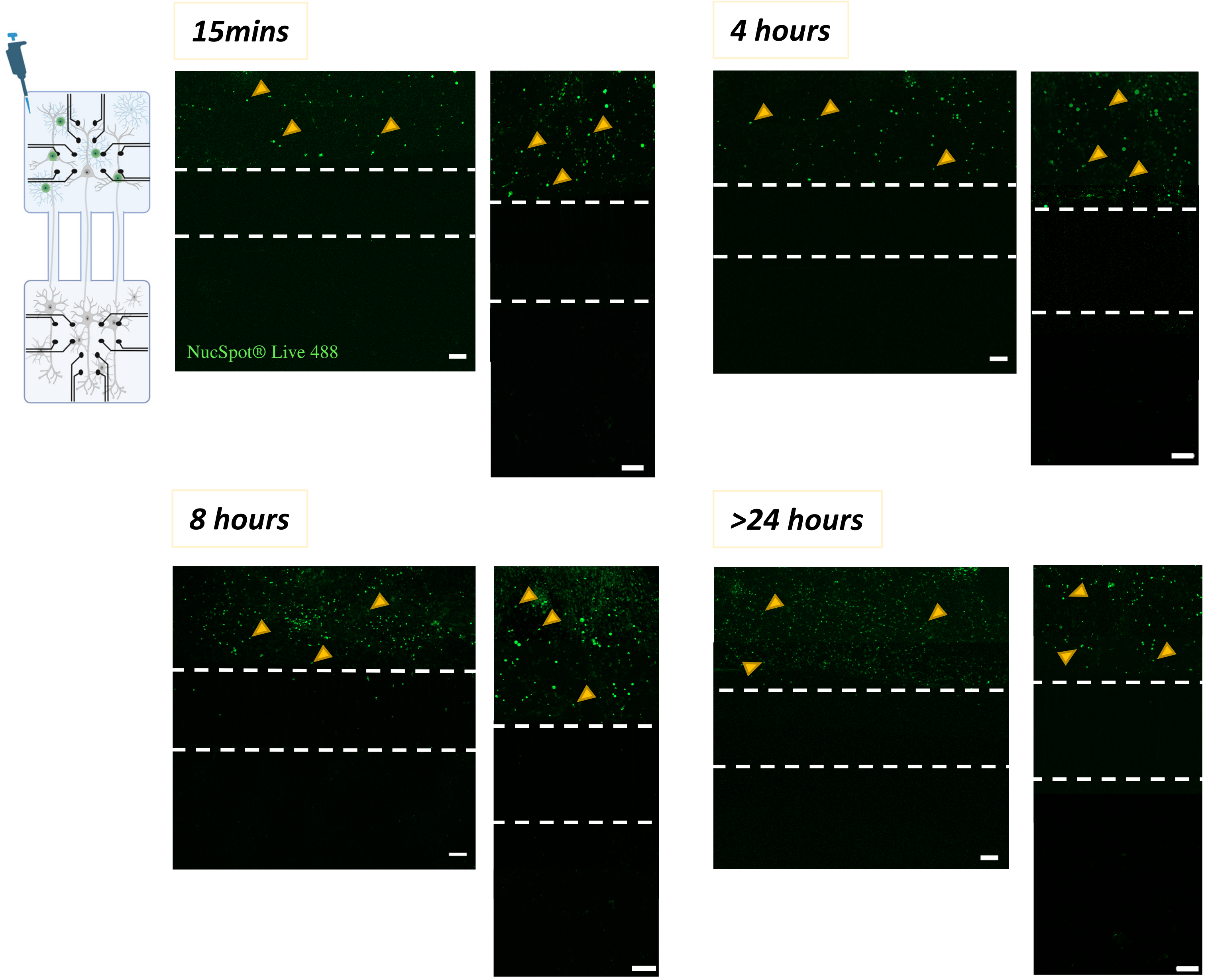

B Subsequent addition at the spinal cord compartment

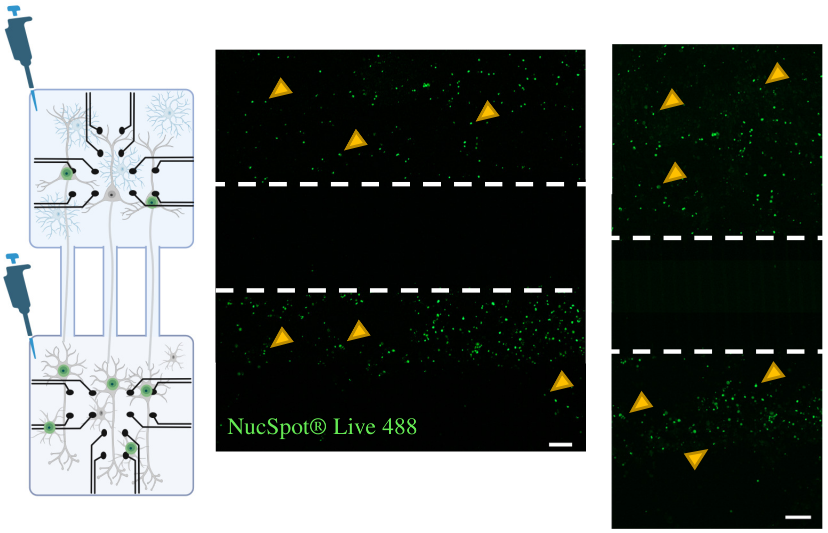

Supplemental Figure 1: Live cell diffusion demonstrates fluidic isolation between compartments related to STAR Methods.

A. Time-lapse imaging of dye diffusion in the microfluidic device following the addition of a live-cell dye to the cortical compartment. Mosaic assembled manually from overlapping confocal fields to represent the broader field of view. Images were captured at 15 minutes, 4 hours, 8 hours, and >24 hours post-addition to monitor diffusion dynamics across the microchannels (n=3 per condition). **B.** Representative mosaic live-cell images of microfluidic devices after dye addition to both compartments, demonstrating expected diffusion patterns (n=3). Scale bars=100µm.

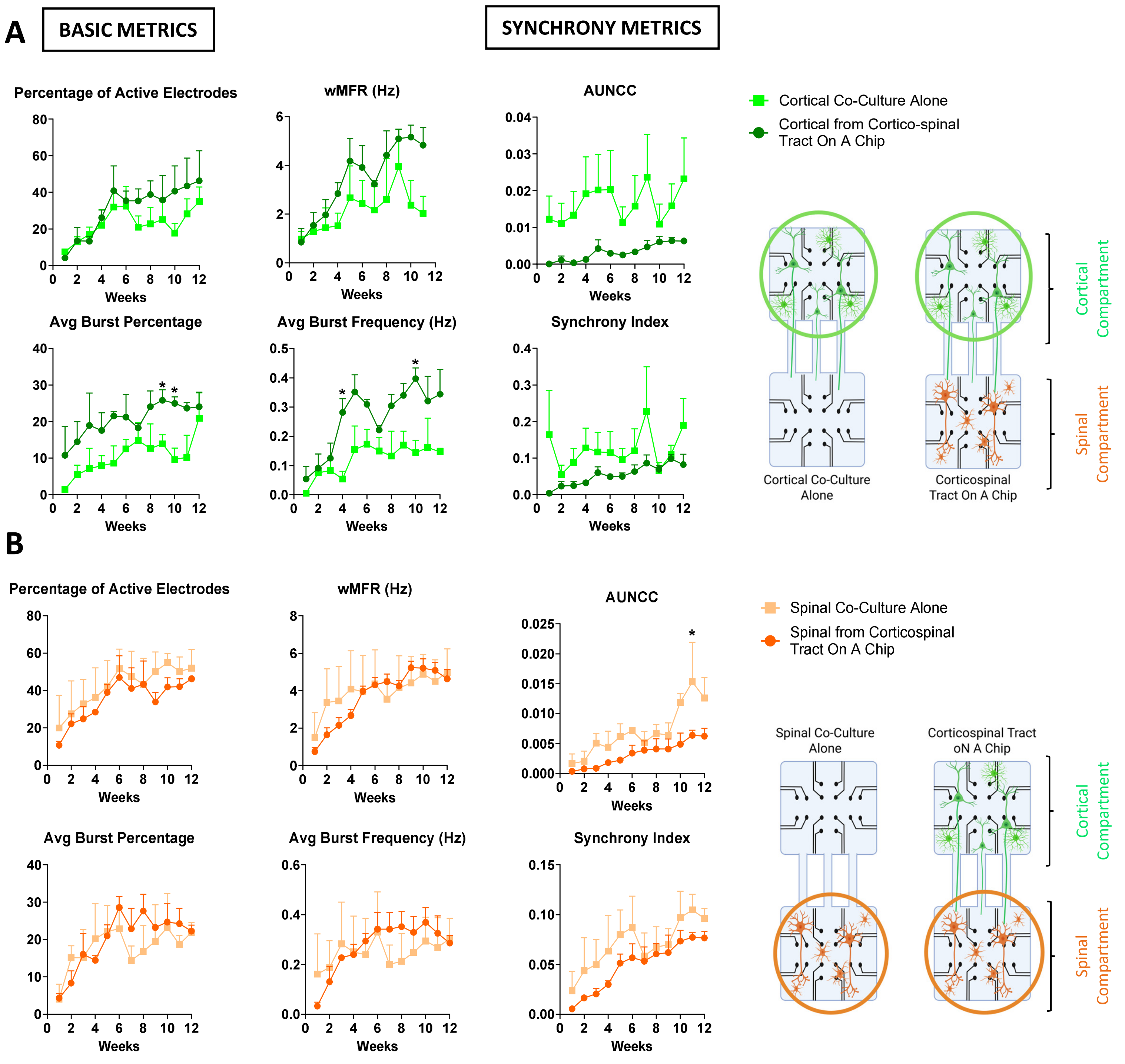

Supplemental Figure 2: Comparison of electrophysiological activity and maturation of single population chips (one neuronal population) with CST-on-a Chip (two neuronal populations), related to Figure 4.

A. CNs and CA single population cultures compared to CST-On-A-Chip setup with both cortical and spinal co-cultures **B.** SpMN and SpA compared to CST-On-A-Chip setup, and illustration of both plating setups (n=8-9 devices on MEA per condition). One-way ANOVA, * $p<0.05$, wMFR: weighted mean firing rate, AUNCC: area under normalized cross correlation.

A

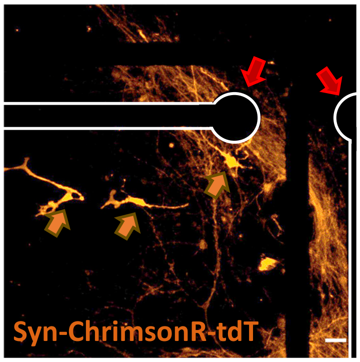

B. Orange (stimulation) VS Green Light (control) stimulation show wavelength –specific response

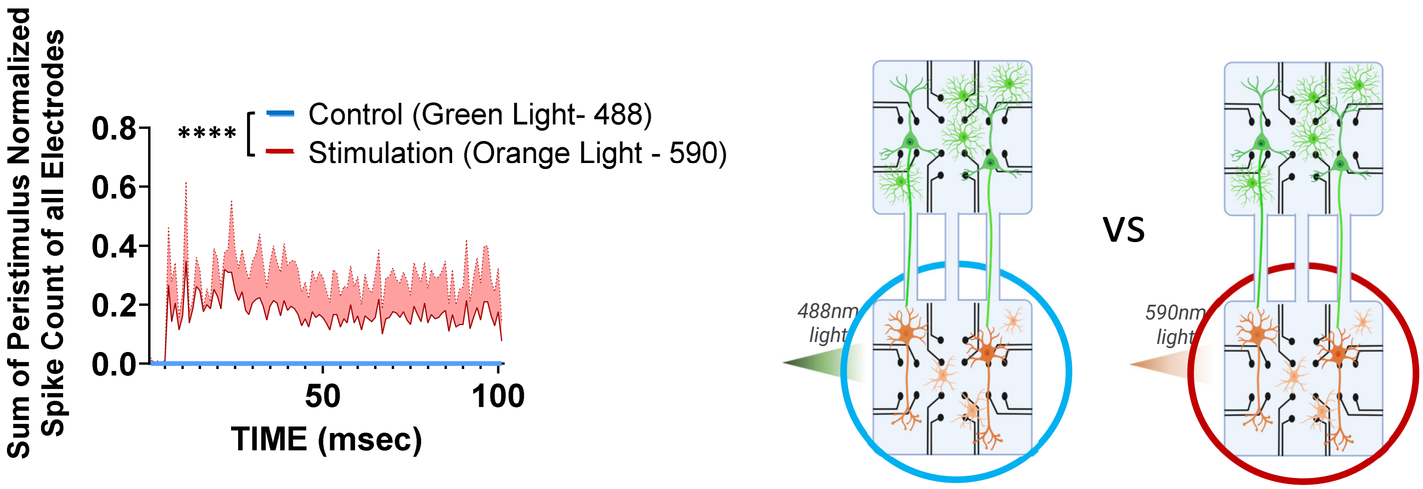

C. Orange light stimulation evokes spinal but not cortical neuronal response

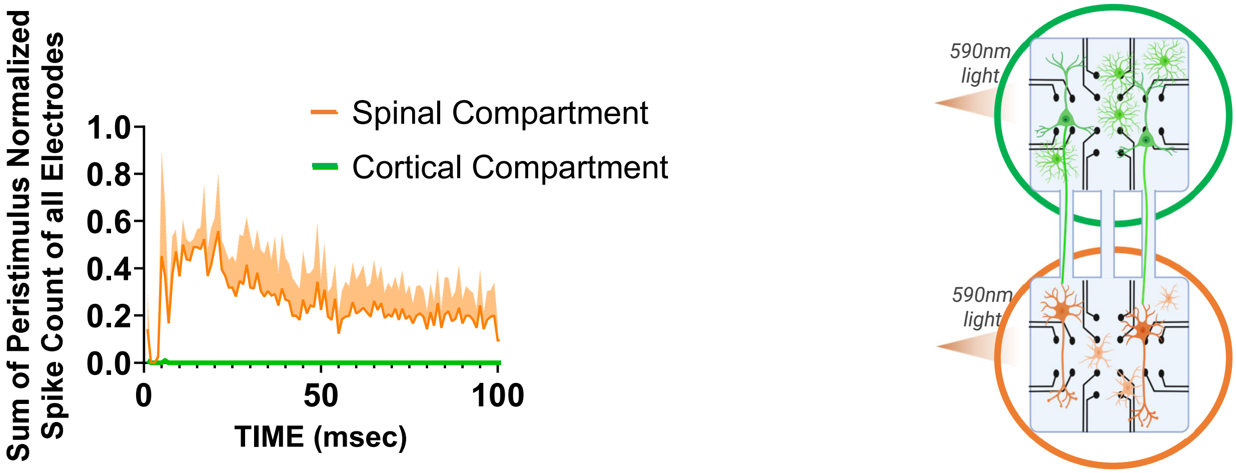

Supplemental Figure 3: Optogenetic activation of SpMN confirms absence of retrograde cortical electrophysiological responses, related to Figure 6.

A. Live Fluorescence image of td Tomato expressed under the synapsin promoter in the spinal compartment (red arrows show electrodes, orange arrows show transduced spinal neurons). **B.** The response recorded from the spinal neurons is specific to orange light stimulation (ChrimsonR-related response), and is significantly higher compared to the control stimulation (green light). **C.** Orange light stimulation activates spinal cord neurons transduced with AAV9-Syn-ChrimsonR-tdT whereas there is no corresponding response from the cortical neurons. Colored circles represent chambers from which data were recorded. Wilcoxon test, **** P<0.0001. Scale bar 50µm.
